# Climate Change From a Distance: An Analysis of Construal Level and Psychological Distance From Climate Change

**DOI:** 10.3389/fpsyg.2019.00230

**Published:** 2019-02-22

**Authors:** Susie Wang, Mark J. Hurlstone, Zoe Leviston, Iain Walker, Carmen Lawrence

**Affiliations:** ^1^School of Psychology, The University of Western Australia, Perth, WA, Australia; ^2^Faculty of Behavioural and Social Sciences, University of Groningen, Groningen, Netherlands; ^3^School of Arts and Humanities, Edith Cowan University, Joondalup, WA, Australia; ^4^School of Psychology, University of Canberra, Canberra, ACT, Australia

**Keywords:** climate change, pro-environmental behavior, psychological distance, construal level theory, time perspective, donation behavior, climate change policy

## Abstract

The public perception of climate change as abstract and distant may undermine climate action. According to construal level theory, whether a phenomenon is perceived as psychologically distant or close is associated with whether it is construed as abstract or concrete, respectively. Previous work has established a link between psychological distance and climate action, but the associated role of construal level has yet to be explored in depth. In two representative surveys of Australians (*N* = 217 and *N* = 216), and one experiment (*N* = 319), we tested whether construal level and psychological distance from climate change predicted pro-environmental intentions and policy support, and whether manipulating distance and construal increased pro-environmental behaviors such as donations. Results showed that psychological closeness to climate change predicted more engagement in pro-environmental behaviors, while construal level produced inconsistent results, and manipulations of both variables failed to produce increases in pro-environmental behaviors. In contrast with the central tenet of construal level theory, construal level was unrelated to psychological distance in all three studies. Our findings suggest that the hypothesized relationship between construal level and psychological distance may not hold in the context of climate change, and that it may be difficult to change pro-environmental behavior by manipulating these variables.

## Introduction

Climate change poses a serious threat to the health, security, and prosperity of all people. Increasing public support for climate policies, and willingness to engage in individual climate action is crucial for directing broader, societal level change (Moser, 2016). The need to engage in sustainable adaptation and mitigation action is growing, and yet among the general public there is widespread apathy and unwillingness to act (Clayton et al., 2014, 2015). While the reasons for a lack of public engagement are wide-ranging and complex (see Castro, 2006; Owens and Driffill, 2008; Gifford, 2011; Fielding et al., 2014 for an overview), a burgeoning body of evidence (reviewed in McDonald et al., 2015) indicates that a key variable is the perception of climate change as a distant phenomenon. “Psychological distance” is a theoretical construct that refers to the subjective perception of distance between the self and some object or event. Several studies have shown that public concern about climate change decreases as perceived psychological distance of climate change from the self increases (Uzzell, 2000; Leiserowitz, 2005; Lima and Castro, 2005; Reser et al., 2012; Spence et al., 2012; Pahl and Bauer, 2013).

Research on this topic has been guided by the construal level theory of psychological distance (CLT; Trope and Liberman, 2010), according to which objects or events that are perceived as psychologically close tend to be construed in a “concrete” manner (yielding a specific representation of those objects or events), whereas objects or events that are perceived as psychologically distant tend to be construed in an “abstract” manner (yielding a broad representation of those objects or events). If people’s perceptions of distance from climate change are governed by a construal level process, then the level at which people construe climate change should be an important determinant of their support for climate action. For example, a concrete construal level may lead climate change to be perceived as psychologically close, which may result in greater acceptance of the problem and willingness to address it; conversely, an abstract construal level may lead climate change to be perceived as psychologically distant, which may result in lower acceptance of the problem and willingness to tackle it.

Understanding the possible link between perceived psychological distance, construal level, and support for climate action is therefore important, since it may provide insights into ways in which climate change communicators can reduce the perceived distance of climate change by manipulating construal level. However, as noted by McDonald et al. (2015) in their review of this field, although much research effort has been expended on understanding the link between the perceived psychological distance of climate change and support for climate action, results have been inconsistent. In three studies, we address this issue by systematically exploring the links between psychological distance, construal level, and support for climate action.

### The Construal Level Theory of Psychological Distance

Psychological distance can be defined as a subjective perception of distance between the self and some object, event, or person. Psychological distance varies as individuals transcend immediate and direct experience, to imagine hypothetical situations, understand socially distant people, care about events in faraway places, and plan for and remember distant times.

The concept of “perceived distance” as a predictor of behavior emerged in the work of Lewin (1951). Lewin’s work on field theory introduced the idea that human behavior may be understood according to distances and forces perceived between the self and other entities. The entities that can affect our behavior include the people that we know, events that occur, values we hold, future goals, past memories, and so on.

More recently, CLT theorists introduced four dimensions of psychological distance that may impact on the self: temporal distance, spatial distance, social distance, and hypothetical distance (Trope and Liberman, 2010). Together, these dimensions describe the “perception of when [an event] occurs, where it occurs, to whom it occurs and whether it occurs” (Trope and Liberman, 2010, p. 442).

According to CLT, greater psychological distance is accompanied by a subsequent increase in mental abstraction. The act of moving beyond immediate experience–such as thinking about the future, considering distant locations or people–occurs through this process of mental abstraction (Soderberg et al., 2015). Any moment that is not part of the immediate experience is at once more distant, and considered in more general terms. The notion of “level of construal”–which refers to whether an object or event is represented abstractly or concretely–originates in categorization theories (Rosch, 1999), where items may be categorized into high-level groups, that focus on abstract, superordinate and central features, such as “chairs,” or to low-level groups, that focus on concrete, specific and peripheral features, such as “wheelchairs” (Trope and Liberman, 2010).

Psychological distance and construal level share the important feature of varying with preferential attention to information. At any point, an individual’s level of abstraction may change, depending on the pertinent goal. High-level construals, and abstraction, may be active over greater temporal distance because the meaning of an abstract construal is unlikely to change, whereas the relevance of a concrete construal may be temporary (Trope and Liberman, 2010). For instance, when asked about health behaviors in the long-term, the salient construal may be “exercise regularly,” but if framed in the short-term, the prevailing construal may be “go for a run before work.” The central idea is that distant entities are construed abstractly, whereas those near are construed more concretely (Trope and Liberman, 2010). Climate change, for instance, if perceived as distant, may predominantly be conceived of in the abstract. The implication is that an abstract and distant perception of climate change produces vague and uncertain conceptualizations of the issue, which may render it difficult to conceive of specific ways to address climate change (Spence et al., 2012).

Past research has found a strong relationship between psychological distance and construal level in the context of general perceptions and cognitions (Trope and Liberman, 2010; Soderberg et al., 2015): construal level shapes judgments of probability (Todorov et al., 2007), temporal and spatial location (Bar-Anan et al., 2007; Hansen and Trope, 2012), and social information (Ledgerwood et al., 2010). In the next section, we review evidence on the link between construal level and psychological distance in the context of climate change.

### Psychological Distance From Climate Change

Spence et al. (2012) conducted the first study to systematically examine perceptions of psychological distance from climate change along all four dimensions proposed by CLT. In a survey of UK residents, Spence et al. (2012) measured participants’ reported psychological distance from climate change: whether it was perceived to be spatially, temporally, socially or hypothetically distant from the self. They tested whether pro-environmental engagement is best predicted by abstract, distant perceptions of climate change, or concrete, close perceptions of climate change. This rationale was motivated by contrasting research suggesting that both types of perceptions and associated construals may predict pro-environmental engagement. Abstractness fosters a goal-centered mind-set and facilitates decision-making and planning for more distant, abstract events (Liberman and Trope, 2008), and enhances self-control (Trope and Liberman, 2010). Therefore, a psychologically distant and abstract mind-set may to lead to actions that adhere to one’s core beliefs (Liberman and Trope, 2008). Conversely, research also suggests that setting specific and concrete goals promotes behavioral engagement (Locke and Latham, 2002; Rabinovich et al., 2009). A concrete construal may promote a psychologically close view (Trope and Liberman, 2010), one that fosters emotional and cognitive engagement with climate change (Van Boven et al., 2010). Hence, making climate change psychologically close and concrete may make the consequences more tangible.

Spence et al. (2012) found that psychological closeness to climate change correlated with greater concern and greater preparedness to reduce energy consumption. However, greater distance along the social distance dimension (viz. the effect of climate change on developing nations) also predicted preparedness to act. This suggests that both psychological closeness and distance can promote pro-environmental action in different contexts.

Crucially, although Spence et al. (2012) theorized about the relationship between psychological distance and construal level, the latter was not empirically measured. This omission makes interpretation of their findings difficult, because although we know that psychological distance is related to perceptions of climate change, we do not know if is also related to construal level. It is possible, for example, that people are motivated to act on climate change when they perceive it in specific, concrete terms, but it does not follow that they perceive it as psychologically close.

Similarly, while several additional studies have found that climate change is perceived as psychologically distant (Spence and Pidgeon, 2010; Scannell and Gifford, 2011; Brügger et al., 2015b), none of these studies has measured psychological distance *and* construal level simultaneously. Although some studies have experimentally induced different levels of construal and shown that this can affect pro-environmental intentions and behaviors (Shwom et al., 2008; Pahl, 2010; White et al., 2011), crucially, they did not measure the resulting construal and perceived psychological distance from climate change.

The result is that there is no way to ascertain from these studies whether pro-environmental actions have been encouraged by a change in perceived distance from climate change, or a change in construal level. Furthermore, without measuring both the change in construal level and psychological distance, it is difficult to know why construal level and psychological distance manipulations sometimes do not produce a change in pro-environmental actions (Shwom et al., 2008; Hart and Nisbet, 2012). These omissions are noteworthy, not only because understanding cognitions of climate change are intrinsically important, but because construal level has been one of the primary devices used to alter psychological distance (Soderberg et al., 2015).

It is possible that climate change is a context in which psychological distance does not always shift in accordance with construal level. If by varying psychological distance we also affect construal level, and vice versa, then making climate change psychologically close should simultaneously increase concrete construals; conversely, increasing abstract construals should simultaneously increase psychological distance. If a change in one necessarily affects the other, then changing abstract construals of climate change may produce contradictory results: for instance, abstract construal can elicit long-term thinking, and greater self-control (Fujita et al., 2006), which may encourage pro-environmental action. On the other hand, abstract construal may also lead to a psychologically distant view which is related to lower concern about climate change. Importantly, if psychological distance and construal level are always matched, this produces a contradiction about how to encourage pro-environmental action. If they operate relatively independently, there is no such contradiction, and we may conceive of construal level and psychological distance as separate pathways to increase pro-environmental action.

A few studies that have examined psychological distance in contexts such as emotional intensity have found that psychological distance and construal level do not directly relate with one another (Van Boven et al., 2010; Williams et al., 2014). There is further evidence to suggest that construal level and psychological distance operate independently in the climate change context, and may constitute separate pathways to climate action. Devine-Wright (2013) argued that the relevance of local climate change effects does not necessarily negate the relevance of distant, global effects; it is possible to conceive of climate change affecting both local (close), and global (abstract) regions simultaneously, and to be concerned about both. Similarly, Rabinovich et al. (2009) argue that it is possible, and even beneficial, to focus on a combination of close and abstract conceptions of climate change. In sum, it is important, both theoretically and practically, to study the unique contributions of psychological distance and construal level to climate change engagement.

### Current Study

The rest of this paper is structured as follows. We report two studies that examined the extent to which climate action is predicted by measures of psychological distance and construal level (Study 1 and 2). To anticipate, the results of these studies revealed an inconsistent pattern: in Study 1, perceived psychological distance from climate change predicted lower support for individual-level pro-environmental behaviors, whereas construal level predicted support for community-level policy support. By contrast, in Study 2 psychological distance no longer predicted support for individual-level pro-environmental behaviors, but there was some evidence that construal level did. Next, we report an experiment (Study 3) that systematically manipulated construal level and perceived temporal distance from climate change in tandem. Contrary to CLT, construal level did not predict pro-environmental behavior, and counterintuitively, greater, rather than lesser, temporal distance to climate change was associated with greater levels of engagement in pro-environmental behavior.

## Study 1

In the context of climate change, the role of psychological distance has not been distinguished from that of construal level. Previous studies have often used construal level as a proxy for psychological distance, assuming an isomorphic relationship which may not hold in the domain of climate change. Accordingly, the primary objective of Study 1 is to examine the relationship between construal level and psychological distance, by measuring both constructs independently, in addition to their relationship with climate change engagement. Such work is necessary to disentangle the role of psychological distance and construal level, as cognitions of climate change, and as predictors of climate change engagement.

To meet this objective, Study 1 measured all four dimensions of psychological distance defined by CLT, in addition to construal level, thereby replicating and extending the work of Spence et al. (2012). We also incorporated two different measures of pro-climate behaviors: (1) a community-level pro-environmental measure, based on support for different carbon emission reduction policies, and (2) an individual-level pro-environmental measure, based on people’s willingness to make sacrifices for pro-environmental choices.

Two hypotheses were tested. The first was that individuals reporting greater psychological closeness to climate change should be more engaged in pro-environmental and climate change-friendly activities. That is, individuals who report low scores on measures of psychological distance should report higher existing engagement in pro-environmental behaviors, and exhibit support for more effective and costly climate policies. The second hypothesis, derived from CLT, concerns the relationship between psychological distance and construal level. According to CLT, individuals who report greater psychological distance should also score more highly on abstract construal, however, research in the climate change context suggests that measures of psychological distance and construal level may not be closely associated.

Finally, an ancillary objective was to compare two self-report scales measuring psychological distance from climate change: an extended version of the scale used by Spence et al. (2012), and a scale used by McDonald et al. (2013). There is currently little consensus in the literature regarding how to measure psychological distance from climate change. Our goal was to establish the degree of correspondence between these two scales, and to determine which better predicts engagement with climate change. We also compared two scales measuring construal level, a commonly used measure called the Behavioral Identification Form (BIF), based on work by Vallacher and Wegner (1989), and a more quantitative method proposed by Krüger et al. (2014), based on Pettigrew’s Category Width measure (1958).

### Methods

#### Participants

An *a priori* power analysis was conducted using G^∗^Power (Faul et al., 2007) to determine the minimum required sample size. Assuming an effect size of 0.29, based on previous work by Spence et al. (2012) and Brügger et al. (2015b), a sample size of 111 would yield a power level of 0.99, at α = 0.05. A total of 218 (114 female) Australian adults recruited by Qualtrics–a survey company specializing in representative Internet surveys–participated in the study. The mean age was 47.35 years (range 18–84), and the median gross annual income bracket was $35,000–49,999 per year. Age, gender, and income groups approximated a representative distribution of Australia’s population, although high income earners were somewhat over-represented (Table 1).

**Table 1:** Sampled distribution for Study 1 compared to Australian population.

		Sample (%)	Population (%)^∗^	Annual income	Sample (%)	Population (%)
Sex	Male	52.5	49.3	<$15,000	12.9	17.16
	Female	47.5	50.7	$15,000–$24,999	12.9	13.49
				$25,000–$34,999	11.06	15.29
Age	18–24	10	12.29	$35,000–$49,999	14.29	15.50
	25–54	55	53.38	$50,000–$74,999	18.43	16.05
	55–64	22	14.74	$75,000–$99,999	11.06	6.92
	65+	13	19.09	>$100 000	19.35	9.83

*^∗^Data has been corrected to exclude the population under 18 years. Population data were obtained from the Australian Taxation Office (2013) and Australian Bureau of Statistics (2014).*

#### Materials and Procedure

The study was executed as a questionnaire using Qualtrics survey software–a web-based survey software tool for the creation of online survey instruments, distribution of surveys, data collection, storage and analysis.

##### Psychological distance 1 (PD1)

The study adapted and extended the questionnaire items used in Spence et al. (2012) to measure psychological distance. The original measure contained 10 items in total: five measured hypothetical distance, one measured temporal distance, and spatial and social distance were each measured by two items. The questions used different response scales and labels. In the present study, we created 18 items measured on a common response scale ranging from 1 (*strongly disagree*) to 5 (*strongly agree*), with greater endorsement reflecting greater psychological distance. The four dimensions of psychological distance specified by Trope and Liberman (2010) - namely temporal distance, spatial distance, social distance and hypothetical distance - were each measured by four items. Additionally, because one’s perceived temporal and hypothetical distance may vary by location and subject, items were created to reflect temporal *and* spatial distance, and temporal *and* social distance (and the same for hypothetical distance). An example of a question is “Climate change will not change my life, or my family’s lives anytime soon” (temporal and social distance).

##### Psychological distance 2 (PD2)

The second measure of psychological distance was taken from McDonald et al. (2013) and used a continuous sliding scale to measure psychological distance along each of the four distance dimensions. An example question is: “When will climate change impacts occur?” Social distance was measured using two separate items, one to measure intimacy (where “close” refers to friends and family), and one to measure similarity (where “close” refers to perceived similarity and dissimilarity from the self). Responses were recorded on a continuous sliding scale ranging from 0 (*right now*) to 100 (*in the very distant future*). Different labels were used for the questions referring to hypothetical, social, and spatial distance. The use of a second measure of psychological distance allows for comparison and assessment of inter-test reliability. The order of presentation of the PD1 and PD2 scales was counterbalanced across participants.

##### Behavioral identification form (BIF)

To verify the compatibility of psychological distance and construal level in the context of climate change, we used the behavioral identification form (BIF; Vallacher and Wegner, 1989), an established measure of construal level (Soderberg et al., 2015). The BIF is a measure of cognitive processing, and assesses whether participants consider issues in an abstract, vague manner, or in a specific, concrete manner. The task involves a series of two-option forced-choice questions distinguishing whether participants construe actions concretely or abstractly. An example of an item would be whether the participant considers “Growing a garden” to be best described by “planting seeds” (concrete construal), or “getting fresh vegetables” (abstract construal).

##### Response category width (RCW)

Recently proposed measures of construal level are more direct, focusing on tracing the cognitive processes elicited by construal level. Theoretically, abstract perceptions should be broad and have a wide confidence interval, whereas concrete perceptions should be more specific and have a narrower confidence interval. Krüger et al. (2014) argue that “response category width” (RCW) is one way of measuring construal level of psychological distance–the more concretely an object is perceived, the narrower the range ought to be (Krüger et al., 2014).

An RCW scale was constructed and adapted from Pettigrew’s Category Width (Pettigrew, 1958) items, which served as the second measure of construal level. There were two main subcategories of construal level for the RCW scale. Six questions addressed construals specifically related to climate change and the environment, and six questions were taken from the original Pettigrew RCW scale, to measure general tendencies toward abstract or concrete construal. An example item is: “According to a study of 100 households, the average shower taken consumes 62 liters of water. What do you think is the most/least amount of water consumed in a single shower?” Participants were presented with four numerical options each for what they perceived as the upper and lower limit. Responses were coded from 0 to 3, in order of proximity to the average value. Greater scores indicate a wider RCW, and more abstract construal. The order of presentation of the BIF and RCW was counterbalanced across participants.

##### Attitude and belief scales

Additional items were included to assess the criterion validity and ability of psychological distance and construal level measures to predict environmental behavior above and beyond known measures. The items related to political identification, views about climate change–including concern about climate change and perceived behavioral control (Leviston et al., 2014)–and climate change skepticism (Whitmarsh, 2011). Belief in anthropogenic climate change was measured using a categorical item asking participants to indicate the statement that best describes their thoughts about climate change: “I don’t think climate change is happening” (deny); “I have no idea whether climate change is happening or not” (don’t know); “I think that climate change is happening, but it’s just a natural fluctuation in Earth’s temperatures” (natural); and “I think that climate change is happening, and I think that humans are largely causing it” (anthropogenic) (Leviston et al., 2014). In addition, the Myths of Physical Nature scale (Price et al., 2014) was used to measure environmental worldviews. This contains two subscales that measure “ductile” and “elastic” environmental worldviews. The former describes the view that the environment is alterable by human actions, whereas the latter describes the opposite view, that the environment is capable of recovering from human actions. The scale demonstrates good predictive validity for pro-environmental intentions (Price et al., 2014).

Two other measures were included to assess criterion validity. The first measure was time perspective, a distance-related variable that strongly predicts environmental behaviors (Milfont et al., 2012; Arnocky et al., 2013). We used the 14-item Consideration of Future Consequences scale (Joireman et al., 2012), which contains items such as “I only act to satisfy immediate concerns, figuring the future will take care of itself” measured on a response scale ranging from 1 (*very uncharacteristic of me*) to 7 (*very characteristic of me*). The second measure was place attachment, a variable that reflects a bond between person and specific spatial locations. As “global attachments”–a feeling of belonging to the entire world–predict environmental action (Devine-Wright, 2013; Devine-Wright et al., 2015), the place attachment scale (Devine-Wright et al., 2015) was included to test the role of local and global attachments, and their relationship with psychological distance and construal level. This scale measures reported sense of belonging to regions of varying distance from the individual, ranging from one’s neighborhood to the entire world, on a response scale ranging from 1 (*no sense of belonging*) to 5 (*very strong sense of belonging*).

##### Dependent measures

To compare the effect of psychological distance on dependent variables of both high and low abstraction, we incorporated two measures, one pertaining to community-level pro-environmental action and the other to individual pro-environmental behaviors, under the assumption that the former may be construed more abstractly, and the latter more concretely. The community-level dependent measure was a set of five emission reduction policy choices, based on scenarios modeled by the Australian Treasury (2013). The options increased in cost ($0, $700, $900, $1,000, and $1,200 reduction to annual national income, per person, in 2020) and effectiveness (0%, 5%, 10%, 15%, and 25% reduction in emissions by 2020). Participants were asked to choose the emission reduction policy they would vote for in a hypothetical referendum. The individual-level dependent measure was adapted from Steg and Vlek (2009), Markle (2013), and Leviston et al. (2014) and consisted of items assessing whether participants would make personal sacrifices for pro-environmental choices. Participants were asked to indicate the likelihood that they would sacrifice time, money, social relationships and effort for pro-environmental choices, products and actions. The response format ranged from 1 (*very unlikely*) to 5 (*very likely*).

### Results

**FIGURE 1:**
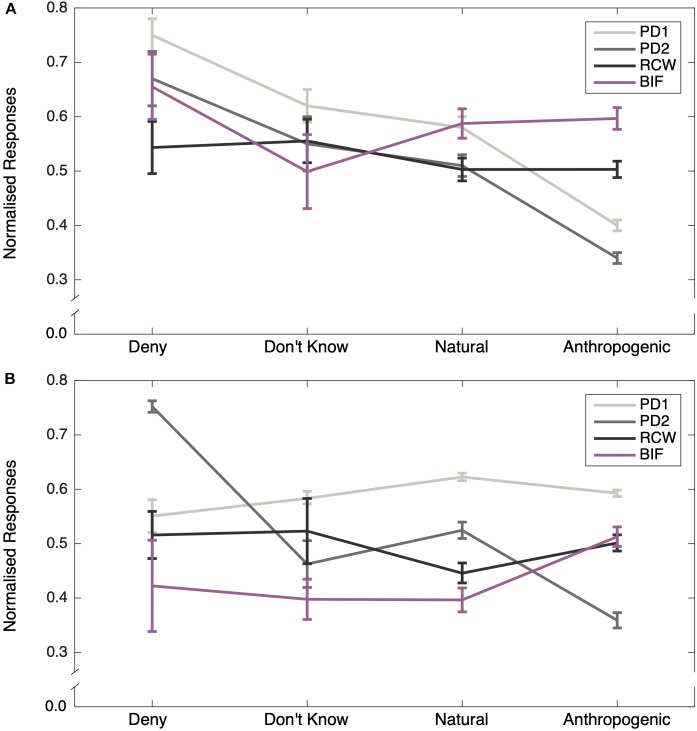
Average responses on the construal level and psychological distance measures as a function of categorical belief in climate change. Panel (**A**) shows the results for Study 1, whereas panel (**B**) shows the results for Study 2. Note that scale scores have been standardized to a 0–1 metric. Error bars represent standard errors.

One participant was removed for selecting the same option for all questions, so the final sample size for analysis was 217. Table 2 shows the descriptive statistics for key variables. Figure 1 shows the responses to measures of psychological distance and construal level, sorted by belief in climate change. Whereas responses on the construal measures do not vary according to climate change belief type, psychological distance appears to decrease with increasing belief in anthropogenic climate change.

**Table 2:** Descriptive statistics for Study 1.

Type of measure	Variable	Minimum (absolute)	Mean	Maximum (absolute)	*SD*	*α*
Covariates	Skepticism	1.00 (1)	2.37	5 (5)	0.96	0.86
	Behavioral control	1.50 (1)	2.99	4.17 (5)	0.43	0.83
	Ductile worldview	1.00 (1)	3.76	5.00 (5)	0.73	0.86
	Elastic worldview	1.00 (1)	2.27	4.28 (5)	0.76	0.85
	Time perspective	1.00 (1)	5.00	6.80 (7)	0.75	0.87
	Place attachment	1.00 (0)	3.52	5.00 (6)	0.97	0.87
Psychological distance	PD1	1.00 (1)	2.43	4.28 (5)	0.83	0.93
	PD2	0.00 (0)	42.10	100 (100)	18.13	0.76
Construal level	BIF	0.00 (0)	0.59	1.00 (1)	0.23	0.85
	RCW	0.11 (0)	1.52	3.00 (3)	0.50	0.79
	Environmental (E)	0.08 (0)	1.53	3.00 (3)	0.54	0.63
	General (G)	0.00 (0)	1.51	3.00 (3)	0.66	0.65
Dependent measures	Individual pro-environmental behavior	1.00 (1)	3.50	5 (5)	0.49	0.69
	Policy choice	1.00 (1)	3.32	5 (5)	1.46	NA

	**Belief-type**			**% of sample**		

	Deny			6%		
Belief in climate	Don’t know			4%		
change	Natural causes			32%		
	Anthropogenic			58%

*PD, psychological distance; BIF, Behavioral Identification Form; RCW, Response Category Width.*

#### Perceived Psychological Distance From Climate Change

**FIGURE 2:**
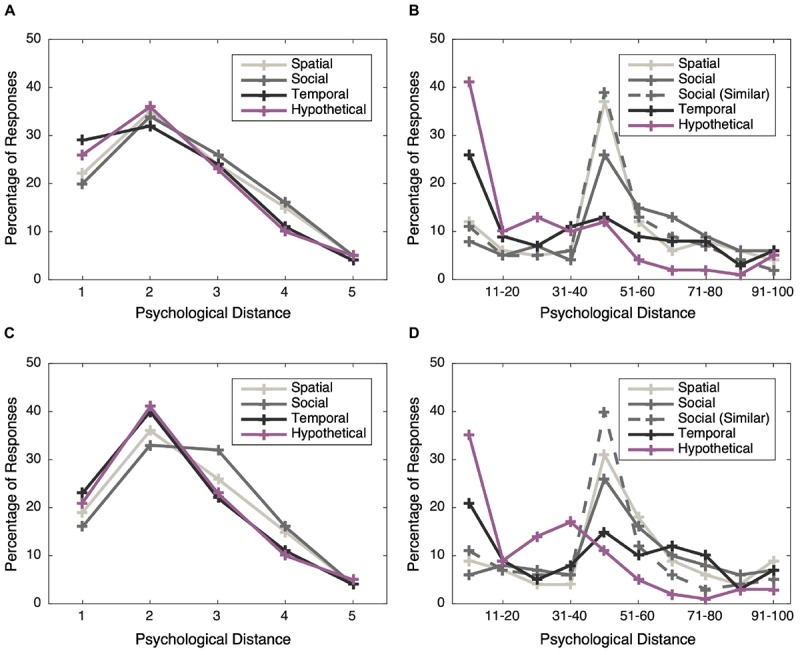
Responses on the psychological distance measures. Left: the percentage of responses, according to response category, on the four psychological distance dimensions indexed by PD1. Greater agreement indicates greater psychological distance from climate change. (**A**) PD1 distributions in Study 1, (**C**) PD1 distributions in Study 2. Right: the distribution of responses according to distance from self, on the five psychological distance dimensions indexed by PD2. In the PD2 scale, the “Social” label refers to intimacy (e.g., friends, family), and “Social (similar) refers to similarity to the self. (**B**) PD2 distributions in Study 1, (**D**) PD2 distributions in Study 2.

Figure 2 shows the percentage of responses given on each of the psychological distance scales. Figure 2A shows PD1, for which the *x*-axis corresponds to codes on the Likert scale from 1 (strongly disagree) to 5 (strongly agree). The distribution of responses was qualitatively similar across all four distance dimensions, with a peak at distance 2 (the “disagree” response option) and a subsequent monotonic decline with increasing distance. For all four distance dimensions, the percentage of responses at distance 1 (strongly disagree) and distance 3 (neither agree nor disagree) were comparable.

Figure 2B shows the pattern of responses on the PD2 scale (responses in this figure have been binned to facilitate graphical illustration of general trends but for subsequent analyses, the aggregated and standardized psychological distance scores were calculated based on the mean of responses). It can be seen from a comparison of Figure 2A,B that responses on the PD2 scale showed a somewhat different profile. Most participants reported that climate change is temporally and hypothetically close (0 distance from the self), but more socially and spatially distant. Many responses for these dimensions were near the midpoint, suggesting that participants may perceive climate change to be neither close nor distant along social and spatial dimensions.

#### Comparing Psychological Distance Scales

Table 3 shows significant correlations between all dimensions of the psychological distance scales, though the degree of relationship between items varied greatly. For the PD1 scale, all dimensions were highly correlated. Similarly, all dimensions of the PD2 scale were correlated, though less highly.

**Table 3:** Correlations for psychological distance in Study 1.

Scale	Distance	1	2	3	4	5	6	7	8	9
PD1	(1) Spatial	–	0.89^∗∗^	0.79^∗∗^	0.83^∗∗^	0.42^∗∗^	0.33^∗∗^	0.66^∗∗^	0.56^∗∗^	0.61^∗∗^
	(2) Social		–	0.76^∗∗^	0.80^∗∗^	0.43^∗∗^	0.32^∗∗^	0.63^∗∗^	0.51^∗∗^	0.52^∗∗^
	(3) Temporal			–	0.87^∗∗^	0.27^∗∗^	0.24^∗∗^	0.74^∗∗^	0.50^∗∗^	0.67^∗∗^
	(4) Hypothetical				–	0.34^∗∗^	0.27^∗∗^	0.65^∗∗^	0.50^∗∗^	0.61^∗∗^
PD2	(5) Social close					–	0.46^∗∗^	0.29^∗∗^	0.50^∗∗^	0.18^∗∗^
	(6) Social similar						–	0.22^∗∗^	0.41^∗∗^	0.18^∗∗^
	(7) Temporal							–	0.54^∗∗^	0.62^∗∗^
	(8) Spatial								–	0.35^∗∗^
	(9) Hypothetical									–

*^∗∗^Correlation is significant at the 0.01 level (2-tailed). *N* = 217.*

##### Principal components analysis

A Principal Components Analysis of the PD1 scale had adequate sampling (KMO = 0.95) and Bartlett’s test of sphericity showed that the null hypothesis could be rejected, χ^2^(153) = 3213.32, *p <* 0.001. An unrotated one-component solution was found, with an eigenvalue of 10.41. All items except one loaded on the first component, which explained 57.89% of the variance (see Supplementary Information). As the PD2 scale only had five items, an individual PCA was not conducted.

##### Correlations with related variables

Correlations between key variables and psychological distance are shown in Table 4. PD1 and PD2 are both positively correlated with climate change skepticism and elastic environmental worldview, and negatively correlated with a ductile environmental worldview, time perspective, and global attachment. That is, greater psychological distance from climate change is associated with greater skepticism, and a view that the environment can recover from any damage caused by humans. Greater psychological closeness to climate change is associated with having an attachment to the world as a whole, having a longer time perspective, and believing that the environment can be altered by human actions. The two measures of psychological distance also correlated positively with one another.

**Table 4:** Correlations for key variables and dependent variables in Study 1.

Variable	1	2	3	4	5	6	7	8	9	10	11	12	13
(1) Skepticism	–	-0.01	-0.67^**^	0.72^**^	0.79^**^	0.62^**^	0.03	0.02	0.04	-0.59^**^	-0.10	-0.50^**^	-0.64^**^
(2) Behavioral control		–	0.19^**^	0.00	-0.05	0.06	0.02	-0.06	-0.09	-0.19^**^	-0.15^*^	-0.09	0.07
(3) Ductile				-0.75^**^	-0.67^**^	-0.57^**^	-0.02	0.00	0.05	0.48^**^	0.10	0.41^**^	0.50^**^
(4) Elastic				–	0.68^**^	0.58^**^	-0.02	0.01	-0.07	-0.53^**^	-0.09	-0.47^**^	-0.51^**^
(5) PD1					–	0.76^**^	0.02	0.01	0.00	-0.57^**^	-0.10	-0.50^**^	-0.53^**^
(6) PD2						–	-0.03	0.07	0.02	-0.45^**^	-0.08	-0.40^**^	-0.38^**^
(7) BIF							–	0.01	-0.06	-0.21^**^	-0.14^*^	-0.15^*^	-0.07
(8) RCW-E								–	0.58^**^	0.02	-0.01	0.06	0.10
(9) RCW-G									–	0.11	0.04	0.11	0.09
(10) Time perspective										–	0.24^**^	0.56^**^	0.53^**^
(11) Place attachment											–	0.18^**^	0.25^**^
(12) PEB												–	0.38^**^
(13) Policy													–

*^∗∗^Correlation is significant at the 0.01 level (2-tailed). ^∗^Correlation is significant at the 0.05 level (2-tailed).*

#### Comparing Construal Level Measures

The RCW measure of construal level consisted of two sections, one section with questions specific to climate change and the environment, and one section with general estimation questions. A Varimax rotated principal components analysis found a two-component solution (KMO = 0.79, Bartlett χ^2^(153) = 655.34, *p* < 0.001), where climate-specific items loaded on one component and general items loaded on a second (see Supplementary Information). As there are no environment or climate related questions in the BIF scale, no PCA was conducted to assess its underlying structure.

As seen in Table 4, the BIF and RCW were not significantly correlated with each other. Particularly noteworthy is the lack of significant correlation between the two construal level measures and the two psychological distance measures. Of the other variables in the study, the BIF correlated positively with time perspective and global place attachment, indicating that a longer time perspective and sense of belonging to the entire world is to some extent linked to abstract construal. RCW had no significant correlations with any variables, except between the two RCW subscales.

#### Predicting Pro-environmental Behavior

Due to high variance inflation factors, we removed a number of variables from the regression (ductile and elastic environmental worldview, see Supplementary Information). The second psychological distance measure (PD2) was initially included, but as it also had a high variance inflation factor score, and did not contribute to the model fit, we removed it from subsequent analysis.

A linear regression was conducted to predict individual-level pro-environmental behavior: willingness to sacrifice time, effort, money and social relationships for the environment. Key variables, including the binary “belief in climate change” variable, were entered. The final model is shown in Table 5, and accounted for 40% of the variance. The model indicates that age, political orientation, behavioral control, time perspective and PD1 were predictors of individual action. Neither construal level measure significantly predicted individual pro-environmental behavior. To test predictors of policy choice, key variables, including the binary “belief in climate change” variable, were used to construct a linear model. The model explained 49.1% of variance (Table 5). Variables influencing the choice of more expensive, effective policies were political orientation, skepticism, behavioral control, RCW-E, time perspective, and place attachment. However, psychological distance as measured by PD1 was a significant predictor of policy choice when climate change skepticism was removed from the model. Inversely, for the individual level behavior, PD1 was a significant predictor, but skepticism was not. When either variable was removed from the model, the other became significant.

**Table 5:** Models predicting policy support and pro-environmental behavior across Studies 1 and 2.

	Pro-environmental behavior				Policy choice			
	Study 1		Study 2		Study 1		Study 2	
Variable	β	*SE*	β	*SE*	β	*SE*	β	*SE*
Gender (M)	-0.05	0.12	-0.26^∗^	0.12	0.19	0.11	0.11	0.14
Age	0.02^∗∗^	0.00	0.01^∗∗^	0.00	0.00	0.00	0.00	0.00
Politics	-0.06	0.05	-0.04	0.04	-0.02	0.04	-0.06	0.05
Income	0.01	0.03	-0.04	0.03	-0.04	0.03	0.02	0.03
Belief	-0.17	0.18	-0.19	0.16	0.26	0.17	0.16	0.18
Skepticism	-0.17^‡^	0.11	-0.14	0.09	-0.41^∗∗∗^	0.11	-0.29^∗∗^	0.10
Behavioral control	-0.05	0.06	0.10	0.07	0.14^∗^	0.05	0.10	0.08
PD1	-0.19^∗^	0.09	-0.09	0.06	0.02^†^	0.08	-0.02^†^	0.07
BIF-E	–	–	0.22^∗∗^	0.08	–	–	0.05	0.09
BIF-G	0.03	0.06	-0.04	0.07	0.03	0.05	-0.03	0.08
RCW-E	0.06	0.06	-0.06	0.07	0.14^∗^	0.06	0.06	0.08
RCW-G	0.01	0.06	0.02	0.07	-0.03	0.06	-0.10	0.08
Time perspective	0.37^∗∗^	0.08	0.25^∗∗^	0.07	0.22^∗∗^	0.07	0.02	0.08
Place attachment	-0.01	0.06	0.10	0.06	0.18^∗∗^	0.06	-0.02	0.07
Constant	-0.37	0.34	0.41	0.30	-0.27	0.31	-0.09	0.34

Observations		217		213		217	213
*R^2^*		0.44		0.41		0.51	0.22	
Adjusted *R*^2^		0.40		0.36		0.48	0.17	
Residual *SE*	0.77	*(df* = 203)	0.80	*(df* = 198)	0.72	*(df* = 203)	0.92	*(df* = 198)

*p < 0.1; ^∗^p < 0.05; ^∗∗^p < 0.01; ^∗∗∗^p < 0.001; ^†^mediated by skepticism; ^‡^mediated by PD1.*

### Discussion

Study 1 investigated the role of psychological distance and construal level in the context of climate change. Following Spence et al. (2012), a principal question was whether psychological distance from climate change predicted environmental behavior. The results show that psychological closeness predicted greater engagement in pro-environmental behaviors, primarily at the individual level.

Psychological closeness predicted individual pro-environmental behavior, over and above the variance explained by variables such as age and political orientation, belief in climate change, skepticism, and behavioral control. Those who reported greater psychological distance were less willing to make individual sacrifices – their time, money, effort and social status – for environmental gains. At the community-level, psychological distance only predicted support for more effective emission reduction policies when climate change skepticism was removed from the model.

As psychological distance and construal level have both been theorized to–and have been empirically shown to–affect one another (Soderberg et al., 2015), it was expected that construal level might also predict willingness to undertake environmental actions. However, the results in this study indicate that construal level predicted engagement in the reverse direction, at least in the case of support for emission reduction policies. That is, those with a more abstract construal of the environment were more likely to support more expensive and more effective emission reduction policies, although this result was not statistically reliable.

The construal level literature suggests that there should be a positive association between psychological distance and abstract construal. This finding has been well substantiated in different fields (Soderberg et al., 2015). However, in the present study there was no evidence that psychological distance increased with abstract construal–construal level and psychological distance were uncorrelated across all measures. Psychological distance and construal level also appear to operate independently–the two variables only jointly predicted one dependent variable (policy choice) and in opposite directions from one another. Not only are psychological distance from climate change and construal of climate change uncorrelated, they also behave qualitatively differently as predictors.

Together, the findings show that psychological distance from climate change (closeness to climate change) predicted individual-level behavior, whereas construal level (abstract construal of climate change) predicted community-level action. The finding that psychological predicts environmental behavior is consistent with that of Spence et al. (2012), but the distinction between predictors of individual and community levels of action is a novel one. People who perceive climate change as a distant issue are less likely to express intentions to mitigate climate change, whereas those who think abstractly about climate change are more likely to support climate action on an abstract level.

A key limitation of the present study is that the construal level measures were not related to one another, which renders it difficult to assess whether they are measuring the same psychological construct. One reason for the lack of relationship may be because the RCW scale has items specifically addressing environmental topics, whereas the BIF scale does not. Further, while RCW-E was a significant predictor of policy choice, given that this is a novel scale, it would be prudent to examine whether this finding generalizes to a new dataset. We sought to address these potential issues in a second study.

## Study 2

To overcome the limitations of Study 1, we conducted a replication using an augmented version of the BIF. The BIF is a recognized measure of construal level, which has been validated in previous studies (Fujita et al., 2006; Soderberg et al., 2015), whereas the RCW is not (although it possesses characteristics that would lead one to expect that it constitutes a viable measure of construal level; see Krüger et al., 2014).

Further, whereas the psychological distance items dealt specifically with climate change, the BIF items measured construal of “general” actions, but did not measure construal of “climate change” or “pro-environmental” actions specifically. In Study 1, we used two putative measures of construal level, namely the BIF (Vallacher and Wegner, 1989) and the RCW scale (Pettigrew, 1958; Krüger et al., 2014), with only the latter measuring environment-related items. Accordingly, it remains possible that the BIF might predict environmental behavior, and perceived psychological distance from climate change, if it contained items measuring construal of climate change directly. To test this possibility, a replication of Study 1 was conducted using an augmented version of the BIF that contained items assessing general and environmental construals.

### Methods

#### Participants, Materials, and Procedure

A total of 216 (105 female) Australian adults recruited once again by Qualtrics.com participated in the study. The mean age was 43.48 years (range 18–79), and the median gross annual income bracket was $35,000–49,999 per year. Age, gender, and income groups approximated a representative distribution of Australia’s population, to the same specifications as Study 1.

The materials and procedure of the study followed that of Study 1. The only difference was the inclusion of an augmented version of the BIF. The new BIF scale consisted of 22 items–11 items from the original scale that focused on general issues (general sub-scale; BIF-G), and 11 items that focused on environmental issues (environmental sub-scale; BIF-E; see Supplementary Information). Participants were asked to select either a concrete or an abstract description for each action. For instance, the behavior “carpooling” could be described as “sharing transportation with others” (concrete), or “reducing the number of cars on the road” (abstract), or the behavior “taking public transport” could be described as “catching a bus or train” (concrete) or “traveling in an energy efficient way” (abstract).

### Results

Table 6 shows the descriptive statistics for key variables. While in Study 1, responses on the two psychological distance measures decreased with increasing belief in anthropogenic climate change, in Study 2 this pattern was only replicated for the PD2 measure, whereas responses on the PD1 measure did not vary according to climate change belief category. However, replicating Study 1, responses on both construal level measures were invariant with respect to climate change beliefs (Figure 1B). The psychological distance measures also exhibited similar distributions to those observed in Study 1 (Figure 2B).

**Table 6:** Descriptive statistics for Study 2.

Type of measure	Variable	Minimum (absolute)	Mean	Maximum (absolute)	*SD*	α
Covariates	Skepticism	1.00 (1)	2.50	5.00 (5)	0.96	0.87
	Behavioral control	1.50 (1)	3.15	5.00 (5)	0.72	0.76
	Ductile worldview	1.00 (1)	3.71	5.00 (5)	0.66	0.79
	Elastic worldview	1.00 (1)	2.47	5.00 (5)	0.78	0.81
	Time perspective	1.00 (1)	4.65	6.93 (7)	0.82	0.80
	Place attachment	1.00 (0)	3.60	5.00 (6)	0.89	0.89
Psychological	PD1	1.00 (1)	2.48	5.00 (5)	0.73	0.94
distance	PD2	0.00 (0)	43.58	100 (100)	17.91	0.73
Construal level	BIF	0.00 (0)	0.46	1.00 (1)	0.20	0.79
	Environmental	0.00 (0)	0.46	1.00 (1)	0.25	0.68
	General	0.00 (0)	0.46	1.00 (1)	0.22	0.63
	RCW	0.00 (0)	1.45	3.00 (3)	0.50	0.78
	Environmental	0.00 (0)	1.49	3.00 (3)	0.55	0.66
	General	0.00 (0)	1.39	3.00 (3)	0.59	0.61
Dependent measure	Individual pro-environmental behavior	1.71 (1)	3.37	4.65 (5)	0.52	0.75
	Policy choice	1.00 (1)	3.07	5 (5)	1.47	NA

	**Belief-type**			**% of sample**		

Belief	Deny			3%
	Don’t know			6%
	Natural causes			35%
	Anthropogenic			56%

#### Comparing Construal Level Measures

Correlations between the environmental form of the BIF and psychological distance, the RCW, and other related variables are shown in Table 7. The BIF-E was positively correlated with behavioral control and ductile worldview, and negatively correlated with PD1, PD2, elastic worldview, and skepticism. The BIF-G showed weaker, but still significant correlations with some of these variables, but no relationship with skepticism, and PD2. The BIF-E and BIF-G were moderately correlated with one another, but notably, neither correlated with the RCW scales. As in Study 1, the RCW items showed no significant correlations with any variables besides themselves (PCA shown in Supplementary Information).

**Table 7:** Correlations for key variables and dependent variables in Study 2.

	2	3	4	5	6	7	8	9	10	11	12	13	14
(1) Skepticism	-0.54^**^	-0.50^**^	0.64^**^	0.74^**^	0.53^**^	-0.31^**^	-0.13	-0.10	-0.05	-0.49^**^	0.05	-0.24^**^	-0.43^**^
(2) Behavioral control	–	0.39^**^	-0.46^**^	-0.55^**^	-0.43^**^	0.39^**^	0.30^**^	-0.03	-0.03	0.50^**^	0.21^**^	0.26^**^	0.31^**^
(3) Ductile		–	-0.57^**^	-0.60^**^	-0.39^**^	0.29^**^	0.26^**^	0.08	-0.01	0.42^**^	0.06	0.37^**^	0.25^**^
(4) Elastic			–	0.66^**^	0.46^**^	-0.35^**^	-0.19^**^	-0.09	-0.02	-0.52^**^	0.01	-0.32^**^	-0.37^**^
(5) PD1				–	0.69^**^	-0.32^**^	-0.22^**^	-0.05	0.00	-0.53^**^	-0.03	-0.30^**^	-0.41^**^
(6) PD2					–	-0.18^**^	-0.11	-0.01	-0.04	-0.33^**^	0.01	-0.22^**^	-0.33^**^
(7) BIF-E						–	0.58^**^	-0.02	-0.08	0.42^**^	0.16^∗^	0.34^**^	0.21^**^
(8) BIF-G							–	0.09	0.07	0.26^**^	0.19^**^	0.14^∗^	0.08
(9) RCW-E								–	0.48^**^	0.04	-0.17^∗^	-0.03	0.06
(10) RCW-G									–	0.09	-0.17^∗^	0.01	-0.09
(11) Time perspective										–	0.11	0.42^**^	0.27^**^
(12) Place attachment											–	0.20^**^	-0.02
(13) Pro-environmental behavior												–	0.14^∗^
(14) Policy													–

*^∗∗^Correlation is significant at the 0.01 level (2-tailed). ^∗^Correlation is significant at the 0.05 level (2-tailed).*

To probe the BIF and RCW scale in more depth, we examined the underlying component structure of these measures, the results of which are given in Supplementary Information. In brief, there were six components extracted. The RCW sub-scale items tended to load on the same components, environmental items loaded on the same components, and general items loaded on the same components. The BIF item loadings fell on three components, apparently distinguished by the nature of the behaviors described, rather than their (lack of) environmental content. For instance, general items such as “greeting someone,” and “resisting temptation,” loaded on the same component as the environmental item “using canvas bags for shopping,” while environmental items such as “recycling,” and “installing solar panels,” loaded on a separate component, with general behaviors such as “measuring a room for carpeting.”

#### Predicting Pro-environmental Behavior

Table 5 shows the results of a linear regression predicting pro-environmental behavior, contrasting Study 1 and 2. The model explained 32.5% of variance. The reliable predictors of pro-environmental behavior in Study 2 were gender, age, political orientation, BIF-E, time perspective and place attachment. Other variables, including psychological distance and skepticism were not significant predictors. There was no replication of the mediation effect found in Study 1 whereby the effect of psychological distance (skepticism) on pro-environmental behavior varied according to whether the skepticism (psychological distance) measure was included or excluded in the regression analysis.

To examine the effect of adding the BIF-E, we conducted the regression in two steps, adding BIF-E at the second step. The contribution of the variable to the model was small but significant (*R*^2^ change = 0.021, *F* = 7.109, *p* = 0.008). The step-wise model is shown in Supplementary Information. However, the introduction of BIF-E did not produce notable differences to the variance attributed to PD1, skepticism, or any of the construal level measures.

For the policy choice variable, the model predicted 19% of variance, and marginally significant predictors were PD1, skepticism and RCW-G. This model explains considerably less variance in the data than in the three previous analyses, despite including the same variables.

### Discussion of Studies 1 and 2

The aims of Study 2 were to replicate Study 1, and to incorporate the role of a new BIF scale corresponding to environmental behaviors (BIF-E). The findings from Study 2 do not replicate the results of the first study; psychological distance was not a predictor of individual pro-environmental behavior, and construal level was not a predictor of community-level policy choice. However, there are some results that, when combined, allow us to piece together a picture of how psychological distance and construal level may operate in the context of climate change.

#### Does Psychological Distance Predict Pro-environmental Behavior?

The finding from Study 1 that psychological closeness to climate change would be associated with greater willingness to act pro-environmentally, was not replicated in Study 2. Psychological distance did not predict pro-environmental behavior at the individual level in the full model, but PD1 was marginally significant when predicting policy choice. One potential factor may be the difference in psychological distance scores by belief type (Figure 1). In Study 1, psychological distance (measured by PD1) was lowest for those reporting belief in anthropogenic climate change, whereas in Study 2, the same group had similar mean scores on PD1 to those in the “deny” and “don’t know” groups.

There also appears to be a lot of shared variance with skepticism. Looking at all four analyses, the variance attributed to PD1 shifted when skepticism was added to the model (Study 1, policy; Study 2, policy), and the variance attributed to skepticism shifted when PD1 was added to the model (Study 1, individual, Study 2, policy). Follow-up mediation analyses (see Supplementary Information) show significant models for PD1 mediating skepticism and vice versa. The correlational design and the inconsistent pattern of results render it difficult to establish a clear relationship between these variables.

#### Does Construal Level Predict Pro-environmental Behavior?

The role of construal level was inconsistent across both studies. The newly added environmental BIF scale was a significant predictor for individual pro-environmental behaviors, but not policy choice. This means that in Study 2, individual pro-environmental behavior was significantly predicted by abstract construal of environmental actions. The addition of BIF-E was an improvement upon the general scale, but considering the finding in Study 1 that abstract construal predicted the abstract behavior, it was expected that the BIF-E would play a role. This was not the case, and further, despite being a significant predictor in Study 1, RCW-E did not play a role in predicting policy choice in Study 2.

One notable aspect of the data is the importance of environment-specific construal level scales: in Study 1, RCW-E predicted policy choice, and in Study 2, BIF-E predicted pro-environmental behavior. The general subscales of both BIF and RCW did not play a role in predicting either. This indicates the potential importance of using topic-specific construal level scales.

#### Does Construal Level Relate to Psychological Distance?

While in Study 1, none of the construal level measures correlated with measures of psychological distance, in Study 2, the BIF construal level measure was correlated with psychological distance and this correlation was stronger for the BIF-E than the BIF-G. However, the correlation is in the opposite direction to what is expected based on CLT, wherein concreteness equates to closeness, and abstractness equates to distance. On the contrary, we find that the BIF and psychological distance are negatively correlated, such that greater abstract construal correlates with less psychological distance. This, combined with the results from the regressions, suggests that construal level and psychological distance do not always correspond, and may represent two separate pathways to environmental action.

One particular limitation of the use of the BIF to measure environmental construal lies in the fact that by definition, abstract construal tends to tap into higher order values, and therefore environmental actions (such as using a shower timer) described *abstractly* tend to contain environmental value orientations (e.g., reducing water use), whereas concrete construals involve lower-order descriptions of actions (e.g., having shorter showers), and typically do not. The result is that abstract answers to items on the BIF-E may contain more explicit environmental aims than concrete answers. The potential separation of these factors is one that is worth considering in future work.

#### Measurement of Psychological Distance

The two studies compared two measures of psychological distance, to ascertain which measure had the greater explanatory power, and to answer theoretical questions about psychological distance. Both PD1 and PD2 scales correlated in the directions expected, with all theoretically related variables, with the exception of construal level. Psychological distance correlated positively with climate change skepticism and elastic environmental worldview, and negatively with ductile environmental worldview and global place attachment.

There is strong evidence to suggest that both PD scales measure the same underlying construct. Aside from possessing the same relationships with several criterion variables, both measures load primarily on one component. Further, PD1 and PD2 correlate highly with one another, as full scales and as separate dimensions. One difference is that PD1 (Spence et al., 2012) appears slightly superior to PD2 (McDonald et al., 2013) in its capacity to explain pro-environmental behavior, and it has consistently higher correlations with related variables. Length of scale is an important factor in these calculations, so we conducted an analysis using the Spearman-Brown prophecy formula (a test of psychometric reliability that predicts reliability as it varies with scale length), which indicated that if PD2 had the same number of items as PD1, the discrepancy in reliability would disappear. However, the estimated correlations, after correction for attenuation, were still higher for PD1 (see Supplementary Information). An additional concern is that the PD2 scale may not have permitted the full scope of responses. In its current form, the scale does not allow for the answer that climate change will affect all regions and people, regardless of closeness or distance from the self.

Another issue in the measurement of psychological distance is the considerable shared variance between psychological distance and skepticism. One explanation may be that the component of psychological distance that relates to hypothetical distance (whether climate change will happen or not) is analogous to skepticism about climate change. However, as the items across dimensions of psychological distance were all highly correlated, the relationship does not seem driven by the hypothetical distance items. Perhaps in terms of predicting behavior, perceiving climate change to be distant may have the same outcome as being skeptical of its existence.

Additionally, the measurement of psychological distance in PD1 and PD2 may be too literal. In line with CLT, feeling distant from an issue may also lead to abstract, vague thoughts about that issue, whereas feeling close might lead to more specific views. Perhaps for those who are distant from climate change, that distance manifests as more of a vague “feeling,” than specific thoughts about when, where, and to whom it will have an effect.

Of the covariates included, time perspective, a distance-related variable, was one of the strongest predictors of policy choice and pro-environmental behavior. The relationship between time perspective and psychological distance was also strong; those who perceive climate change as psychologically close are also more likely to give greater consideration for future consequences, and be less swayed by immediate rewards. These results support and add to the extensive body of work linking time perspective and pro-environmental action (Milfont et al., 2012; Arnocky et al., 2013). The present findings contribute to this literature by suggesting a possible explanation for this relationship–the effect of time perspective on environmental actions may be explained by an underlying similarity between time perspective and psychological distance. Those with longer time perspectives perceive a stronger connection between present actions and future consequences (Joireman et al., 2012), which is an act of reducing distance between the present and the future. Time perspective may be conceived of as the reduction of psychological distance between now and the distant future. This explanation is consistent with research on temporal discounting, which has shown that in general, people discount future environmental costs (Hardisty and Weber, 2009).

#### Potential Limitations

There may have been external changes that affected the results between the two studies, and particularly the perception of climate change policies. Study 1 was conducted in 2014, when a climate change policy was being changed. Specifically, an Emissions Trading Scheme was being repealed and replaced with a new policy, and so the issue was at the forefront of many political and policy discussions. By 2016, when Study 2 was conducted, this was no longer the case and climate policy was no longer under the spotlight. We can see from a frequency plot of policy support between the two samples that the earlier sample was more supportive of stronger emissions reduction policies (see Supplementary Information).

A separate issue is that Studies 1 and 2 are both correlational, so causality of the observed relationships (e.g., between psychological distance and pro-environmental action) are unclear. It may be that those who are psychologically close make more pro-environmental sacrifices, but it may also be that those less willing to make sacrifices push climate change away psychologically. The latter possibility is consistent with a motivated cognition approach (Hart and Nisbet, 2012; Leviston et al., 2014).

Further, while these correlational results have implications for CLT, particularly for its use in the context of climate change, the lack of relationship in measurement does not suggest a lack of relationship upon manipulation. Individuals are capable of both abstract and concrete construals of climate change, depending on the salient context. As Studies 1 and 2 did not provide a frame, or point of focus for construal level, it is possible that construal level was not salient. This limitation is substantiated by the finding that the effect sizes for the relationship between construal level and psychological distance are larger with greater cognitive engagement (Soderberg et al., 2015).

In Study 3 we experimentally manipulated construal level of, and psychological distance from, climate change to assess whether a relationship exists in a more cognitively engaging context, and whether causal relationships can be established.

## Study 3

The main aim of Study 3 was to manipulate both psychological distance and construal level frames of climate change, and test their role in predicting pro-environmental action in an experimental context. As time perspective was a key variable in the previous two studies, and an important dimension in climate change action, the present study manipulated psychological distance using variations in temporal distance.

Experiments conducted outside the context of climate change tend to show consistent and robust effects: manipulating construal level affects temporal distance, and vice versa (Trope and Liberman, 2003; Soderberg et al., 2015), although most studies only looked at timespans of less than a year. In the environmental context, the findings are less clear. In one case, researchers have found exactly what CLT would predict, namely that shifting a temporal horizon to appear closer increases pro-environmental behavior via concrete construals (Bashir et al., 2014). The aforementioned study manipulated the perceived temporal distance of a future date (e.g., “2020”) by asking participants to mark the year on a horizontal line. The endpoints of the line began in the current year at the time of testing (2010), and ended either in 2025 (2020 future seems distant) or 2085 (2020 future seems close). Bashir et al. (2014) found that the manipulation successfully led participants to feel temporally closer to 2020, and that this predicted intentions and reported environmental behavior. Further, the relationship between temporal closeness and reported behavior was mediated by concrete construals of pro-environmental actions.

However, the effects reported in Bashir et al. (2014) were small and not representative of the general pattern observed in the wider literature. For instance, Rabinovich et al. (2010) conducted a study in which time perspective was manipulated, and found that people intended to behave more in line with their pro-environmental attitudes when envisioning a temporally distant situation, rather than a temporally close one. Roh et al. (2015) found the opposite; that temporal distance (discussing future consequences of climate change) led to reduced action among Republican participants. In other studies, manipulating the future timing of climate impacts has had no effect in some cases (Sundblad et al., 2011), and in other cases produced results that are variable and difficult to explain (Rickard et al., 2016). The latter study manipulated onset of major climate impacts in a close (New York) versus distant location (Singapore), at three future time points (2020, 2047, and 2066). They found that liberals showed less variability in response to manipulations, but that the highest support for climate policy was from conservatives when climate impacts were spatially close, and temporally distant (New York in 2066).

To further complicate matters, methods to evoke the perception of psychological distance can also take the form of framing tasks that seek to induce a particular construal level, confounding the two variables. One well-tested manipulation of construal level is the “how/why” method (Liberman et al., 2007; Hansen and Trope, 2012; Soderberg et al., 2015). It has been shown that framing a task in terms of “how” one might engage in pro-environmental behaviors leads to more concrete construals than framing a task in terms of “why.” Pahl (2010) studied the effect of how/why framing on behavioral intentions for reducing plastic bag usage. Participants estimated engaging in the behavior sooner when they were asked “how” they might reduce plastic bags, rather than when they were asked “why” they might want to reduce plastic bags. This suggests that construal level affects participants’ likelihood of engaging in pro-environmental behavior, specifically in the temporal dimension. If the relationship postulated by CLT holds, we expect that this manipulation will affect both construal level and psychological distance, and that a concrete construal (“how”) will lead to perceptions of psychological closeness, and an abstract construal (“why”) will lead to perceptions of psychological distance.

As discussed earlier, individual difference studies rarely measure both psychological distance and construal level and this problem is also true of the experimental literature on this topic. Additionally, those studies that have experimentally induced different levels of construal to selectively influence pro-environmental behaviors (Shwom et al., 2008; Pahl, 2010) have not measured construal level or psychological distance post-manipulation. Accordingly, in these studies there was no way to verify whether pro-environmental actions have been encouraged by a change in psychological distance, or construal level, or both variables.

The purpose of Study 3 is to plug this experimental gap by co-manipulating psychological distance and construal level. By manipulating the temporal closeness of climate change, and asking participants to evaluate the stimuli either abstractly or concretely, in Study 3 we test the effects of both variables, and measures the corresponding effect on psychological distance and construal level using verified measures. According to CLT, the closest condition should be the one that places climate change at the closest temporal moment, and where concrete construals elicit a sense of psychological closeness. Following this logic, the most distant condition should be the one that situates climate change furthest in the future, and elicits a distant mindset through abstract construals.

Conversely, the findings of Study 1 and 2 suggest that rather than concrete construals, abstract construals tend to predict climate change action. Based on these findings, an alternative prediction would be that the abstract conditions would be more effective than concrete conditions at increasing pro-environmental action.

### Methods

#### Participants

A total of 320 undergraduate students (62% female, mean age = 20.83, *s* = 7.08, range = 17–68) from the School of Psychological Science at the University of Western Australia took part in the study in Perth, in exchange for course credits.

#### Design

The study adopted a 2 (construal level: concrete vs. abstract) × 3 (time horizon: past vs. present vs. future) between-participants design. An additional control condition was included in which participants were not exposed to either the construal level or time horizon manipulations. Participants were allocated at random to the seven resulting between-participant conditions. The total number of participants in each of the seven conditions was as follows: control (*N* = 46), concrete/past (*N* = 47), concrete/present (*N* = 43), concrete/future (*N* = 47), abstract/past (*N* = 45), abstract/present (*N* = 48), and abstract/future (*N* = 44).

#### Materials and Procedure

**FIGURE 3:**
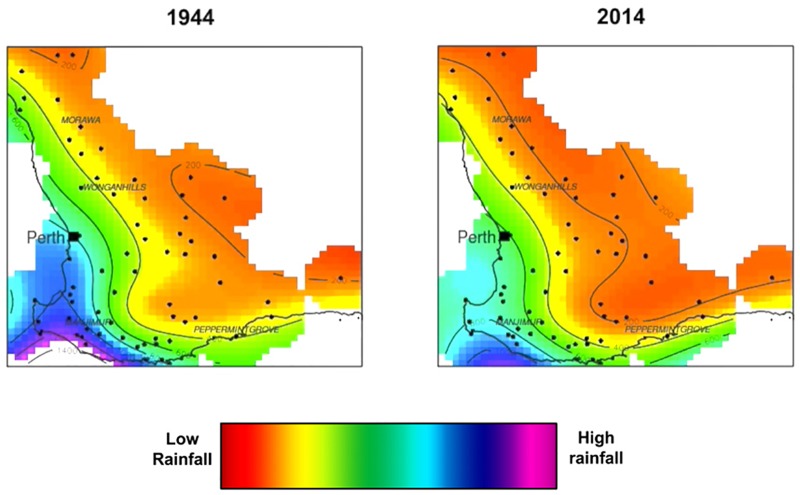
Screenshots of the beginning and end of the videos shown to participants in Study 3. The left image shows rainfall levels at the earliest time point, and the right image shows rainfall levels at the latest time point. Years at the top were altered to fit each condition, and advanced as the video progressed. The scale at the bottom was shown to participants before viewing the video.

Participants were tested individually. They read an information sheet and provided informed consent, after which they were assigned to closed testing rooms fitted with a PC and monitor. In the experimental conditions, participants were shown a video about rainfall in Western Australia–screenshots of which are shown in Figure 3. The video was developed by the CSIRO Climate Adaptation Flagship based on real rainfall data collected between the years 1940 and 2010, and provides a clear visual example of rainfall reduction over time superimposed onto a map of south-west Western Australia, including Perth and surrounds.

The video was altered slightly for different conditions. In the “past” condition, the dates on the video were altered to show the last 70 years. It depicted recorded amounts of rainfall (mm) in the May-June-July period for each year from 1944 to 2014 on a map of Western Australia. This condition placed the onset of severe climate change-linked drought at 2014, and was the condition closest to the year in which the study was conducted (2015). In the “present” condition, the years on the video were modified to show the 70 years around which 2015 was the midpoint (1980 to 2050). This condition placed climate change-linked drought at 2050, and was temporally more distant. In the “future” condition, the years were modified to show the next 70 years, depicting 2015–2085, and placing the onset of climate change-linked drought at 2085.

In all experimental conditions, the video was followed by a message, modified to fit each condition: “Climate change is happening now. There are more dry days now than there ever have been. Due to a persistent decline in rainfall (past condition: *over the last 70 years*; present condition: o*ver the last 35 years, and that will occur over the next 35 years*; future condition: *that will occur over the next 70 years*), Perth dams (past condition: *have received*; present and future conditions: *will receive*) up to 40 percent less water.”

Participants were then asked to respond to attention checks, before completing three questions designed to manipulate construal level using the how/why method (Liberman et al., 2007; Hansen and Trope, 2012; Soderberg et al., 2015). In the concrete construal condition, participants were asked to write responses to questions such as, “How are rainfall patterns changing in Western Australia?” and in the abstract construal condition, participants were asked questions such as “Why are rainfall patterns changing in Western Australia?” Participants in the control condition were not shown the video or accompanying messages, and were not asked to answer construal level questions. All participants were asked to respond to measures of psychological distance (PD1) and construal level (RCW and BIF). The RCW and BIF scales both contained general construal items and environmental construal items.

Next, participants were presented with the first behavioral measure. They were given an endowment of $10, in single $1 coins placed in an envelope on the desk in front of them. In the privacy of their individual testing room, participants had the option of keeping the entire $10, or donating some, or all, of it to Gondwana-Link, a real charity aiming to restore the natural wildlife and landscape in Western Australia. Participants were given a booklet explaining the charity and its purpose, and were invited to explore the website. The box for donations was an opaque locked money box with a coin slot, with coins already placed inside to imply to the participant anonymity of donation. The additional coins were planted by the experimenter, and were not $1 coins, so that the experimenters could distinguish donations.

Participants were then asked to complete a questionnaire measuring demographic variables, and scales used in Study 1 and 2 (time perspective, climate change belief, climate change skepticism, and perceived behavioral control, and a second behavioral measure of pro-environmental behavior: willingness to expend effort and time for the environment).

The final behavioral measure was unobtrusive. As participants were debriefed, they were offered either a Fairtrade chocolate (AlterEco), or a non-Fairtrade chocolate (Lindt) and their chocolate choice was recorded by the experimenter after the participant left the laboratory. A preference for Fairtrade products has been found to be a predictor of global identification (Reese and Kohlmann, 2015) and subsequently pro-environmental intentions and behavior (Devine-Wright, 2013).

### Results

**FIGURE 4:**
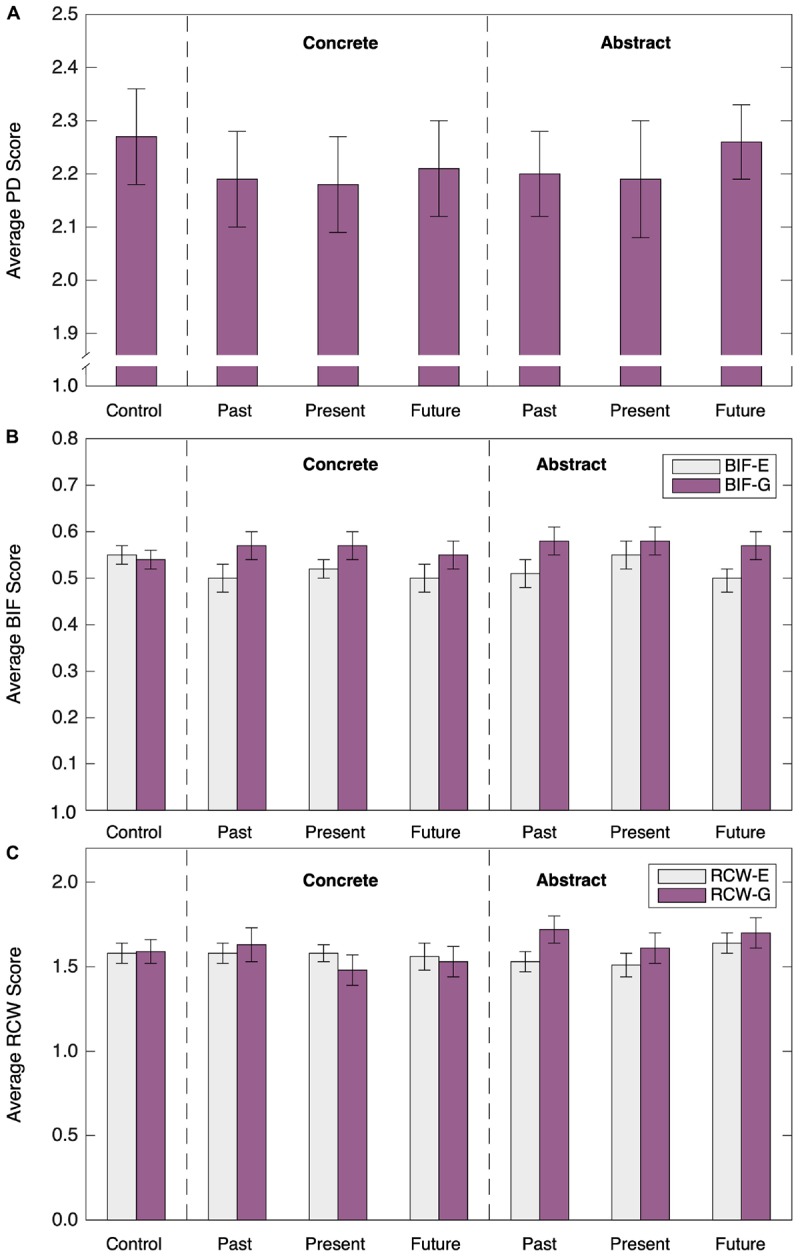
Average PD (**A**), BIF (**B**), and RCW (**C**) scores by condition in Study 3. Error bars represent standard errors.

The final sample included 319 participants. One participant was excluded for failing attention and speeding checks. Descriptive information for measured variables, collapsed across conditions, are shown in Table 8, and PD, BIF and RCW scores are shown in Figure 4.

**Table 8:** Descriptive statistics for Study 3.

Type of measure	Variable	Minimum (absolute)	Mean	Maximum (absolute)	*SD*	α
Covariates	Skepticism	1.00 (1)	2.07	4.20 (5)	0.69	0.77
	Behavioral control	1.50 (1)	3.43	5.00 (5)	0.63	0.72
	PD	1.00 (1)	2.21	3.86 (5)	0.59	0.72
	Time perspective	2.87 (1)	4.79	6.67 (7)	0.78	0.86
Construal	BIF	0.00 (0)	0.54	1.00 (1)	0.15	0.51
level	Environmental	0.00 (0)	0.52	1.00 (1)	0.18	0.33
	General	0.00 (0)	0.57	1.00 (1)	0.20	0.53
	RCW	0.33 (0)	1.58	2.61	0.39	0.72
	Environmental	0.17 (0)	1.57	2.67	0.39	0.56
	General	0.00 (0)	1.61	3.00	0.59	0.56
Dependent	Pro-environmental	1.77 (1)	3.27	4.29 (5)	0.48	0.64
measures	behavior					
	Donation behavior	$0.00 ($0)	$6.03	$10.00 ($10)	$3.98	NA

	**Belief type**			**% of sample**		

Belief	Deny			0.3%
	Don’t know			1.9%
	Natural causes			16.3%
	Anthropogenic			81.3%		

*^∗^All variables were measured after manipulation.*

#### Psychological Distance and Construal Level

The short-form of the PD1 scale (PD) had satisfactory internal consistency reliability (α = 0.72) and the reliability coefficients did not vary appreciably across the different conditions. Figure 4A shows mean responses on the PD measure. To test whether mean scores differed between conditions, we dropped the control condition and conducted a 2 (construal level: concrete vs. abstract) × 3 (time horizon: past vs. present vs. future) ANOVA on the PD scores. There was no significant main effect of construal level, *F*(1,267) = 0.02, *p* = 0.89, no significant main effect of time horizon, *F*(2,267) = 0.22, *p* = 0.80, and no significant interaction between the two variables, *F*(2,267) = 0.60, *p* = 0.55.

The BIF scale was moderately internally consistent (BIF α = 0.51), but with BIF-E (α = 0.33) showing less consistency than BIF-G (α = 0.53). The low alpha was not a result of any single item. The alpha scores were relatively stable in all experimental conditions, except the control condition (BIF-E α = -0.57, BIF-G α = 0.06; for further information see Supplementary Information). Figure 4B shows mean responses on the BIF-E and BIF-G as a function of the different conditions. These data were subjected to a 2 (construal level) × 3 (time horizon) × 2 (BIF: BIG-E vs. BIF-G) ANOVA. There was a significant main effect of BIF, *F*(1,267) = 14.34, *p* < 0.001, η^2^ = 0.05, with larger scores on the BIF-G than the BIF-E, but no significant main effect of construal level, *F*(1,253) = 0.73, *p* = 0.39, and no significant main effect of time horizon, *F*(2,253) = 0.05, *p* = 0.78. All of the two-way interactions and the three-way interaction were non-significant (all *F*s < 1, all *p*-values > 0.4).

The internal consistency of the RCW scale was satisfactory (α = 0.72), although this reliability was attenuated when the internal consistency of the two sub-scales was calculated separately (α = 0.57 for RCW-E, α = 0.60 for RCW-G). The average scores on the two versions of the RCW scale, as a function of the different conditions, can be examined in Figure 4C. These data were once again entered into a 2 (construal level) × 3 (time horizon) × 2 (RCW: RCW-E vs. RCW-G) ANOVA. There was no significant main effect of construal level, *F*(1,267) = 1.34, *p* = 0.25, no significant main effect of time horizon, *F*(2,267) = 0.70, *p* = 0.50, and no significant main effect of RCW, *F*(1,267) = 1.90, *p* = 0.17. However, there was a significant construal level × RCW two-way interaction, *F*(1,267) = 4.96, *p* < 0.05, η^2^ = 0.02, which arose because there was no effect of construal level on the RCW-E scale, *F*(1,267) = 0.09, *p* = 0.76, but responses on the RCW-G scale were higher in the abstract construal condition than in the concrete construal condition, *F*(1,267) = 3.28, *p* = 0.07. The remaining two-way interactions and the three-way interaction were all non-significant (all *F*s < 1.43, all *p*-values > 0.24).

The last measure we examined as a proxy of psychological distance was time perspective. It is plausible that time perspective would be affected by the temporal manipulations. To this end, we conducted a 2 (construal level) × 3 (time horizon) ANOVA on the time perspective scores. Consistent with the earlier psychological distance analysis, there was no significant main effect of construal level, *F*(1,267) = 0.03, *p* = 0.85, and no significant main effect of time horizon, *F*(2,267) = 0.89, *p* = 0.41, but the interaction between the two variables fell just short of conventional significance levels, *F*(2,267) = 2.94, *p* = 0.06, η^2^ = 0.02.

#### Correlations

Correlations between key variables are shown in Table 9. As these variables were measured post-manipulation, interpretation should be made with caution. Consistent with the findings of Study 1 and 2, psychological distance was positively correlated with skepticism, and negatively correlated with behavioral control, time perspective, pro-environmental behavior, and donations. There was no correlation between psychological distance and any of the construal measures.

**Table 9:** Correlations between variables in Study 3.

	2	3	4	5	6	7	8	9	10
(1) PD	-0.07	0.00	-0.03	-0.05	0.59^**^	-0.42^**^	-0.31^**^	-0.36^**^	-0.22^**^
(2) RCW-E	–	0.44^**^	-0.07	-0.07	-0.04	0.01	-0.10	-0.10	0.06
(3) RCW-G		–	-0.12^∗^	-0.08	0.03	-0.07	-0.12^∗^	-0.04	-0.02
(4) BIF-E			–	0.18^**^	-0.04	0.14^∗^	0.15^**^	0.15^**^	0.00
(5) BIF-G				–	-0.05	0.11^∗^	0.30^**^	0.26^**^	0.06
(6) Skepticism					–	-0.35^**^	-0.26^**^	-0.21^**^	-0.19^**^
(7) Behavioral control						–	0.35^**^	0.41^**^	0.21^**^
(8) Time perspective							–	0.42^**^	0.19^**^
(9) Pro-environmental behavior								–	0.18^**^
(10) Donation									–

*^∗∗^ Correlation is significant at the 0.01 level (2-tailed). ^∗^Correlation is significant at the 0.05 level (2-tailed).*

The RCW scale was largely only correlated with itself, although the RCW-G scale was weakly negatively correlated with the BIF-E. A detailed analysis by condition showed that this correlation was only significant in two conditions: concrete/future, *r*^2^ = -0.30, *p* = 0.05, and abstract/future, *r*^2^ = -0.35, *p* = 0.02. In the concrete/future condition, the environmental subscale of RCW was also negatively correlated with the BIF-E, *r*^2^ = -0.34, *p* = 0.02.

The BIF measures partially replicated the findings of Study 2, where BIF-E showed positive correlations with key variables (behavioral control, time perspective, and pro-environmental behavior). In this study, the BIF-G was also positively correlated with the same variables, and more strongly. None of the construal level measures correlated with donation behavior.

#### Predicting Pro-environmental Behavior

**FIGURE 5:**
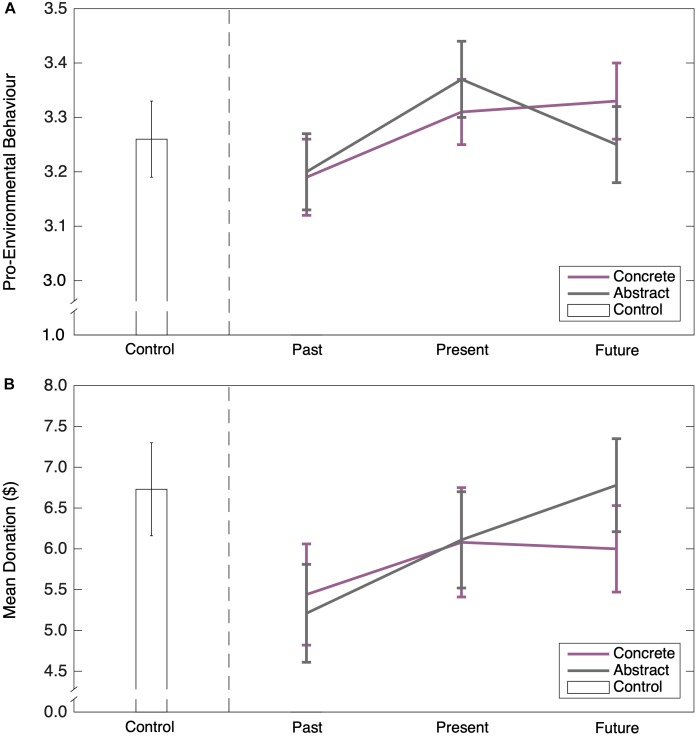
Average PEB scores **(A)** and charity donations **(B)** as a function of condition. Error bars represent standard errors.

Figure 5A shows willingness to engage in pro-environmental behaviors by condition. As above, a 2 (construal level) × 3 (time horizon) ANOVA was conducted on these data. There was no significant main effect of construal level, *F*(1,267) = 0.01, *p* = 0.94, a marginally significant main effect of time horizon, *F*(2,267) = 2.37, *p* = 0.09, with participants in the past condition having lower pro-environmental behavior scores than those in the present and future conditions, and no significant interaction between the two variables, *F*(2,267) = 0.52, *p* = 0.60.

#### Donation Behavior

The donations to Gondwana-Link across the different conditions are shown in Figure 5B. These data were once again analyzed via a 2 (construal level) × 3 (time horizon) ANOVA. There was no significant main effect of construal level, *F*(1,250) = 0.14, *p* = 0.71, no significant main effect of time horizon, *F*(2,250) = 1.18, *p* = 0.31, and no significant interaction between the two variables. Although the main effect of time horizon was not statistically reliable, it merits comment that inspection of Figure 5B reveals a similar trend to that of behavioral intentions, whereby participants in the past condition tended to register lower donations than in the present and future conditions.

#### Chocolate Choice

**FIGURE 6:**
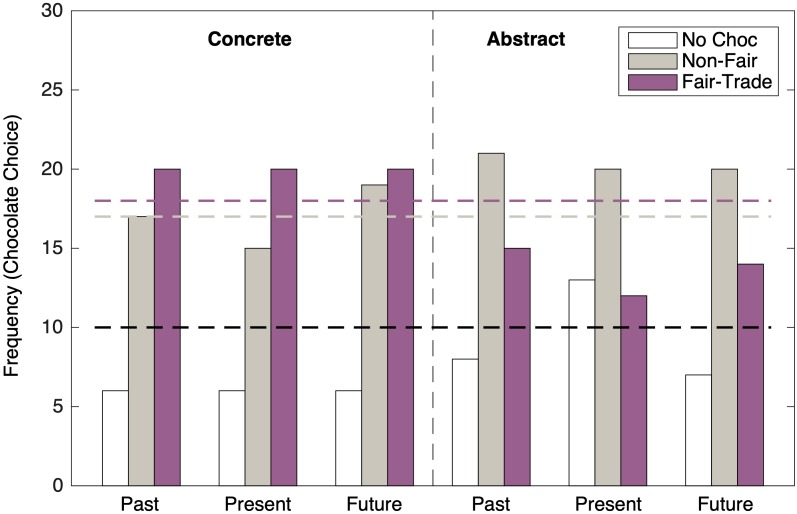
Chocolate choice as a function of condition in Study 3. The broken horizontal lines represent the frequency of “No Choc” (black line), “Non-Fair” (gray line), and “Fair-Trade” (purple line) chocolate choices in the control condition.

Figure 6 shows the chocolate choice data for the different conditions. These data were subjected to a multinomial logistic regression analysis, with chocolate choice (no chocolate vs. non-Fairtrade vs. Fairtrade) as the outcome measure (with “Fairtrade” as the reference category) and construal level, time horizon, and the construal level × time horizon interaction as predictors. A test of the model against a constant only model was not statistically significant, indicating that the predictors as a set did not reliably distinguish between people who chose no chocolate, a non-Fairtrade chocolate, and a Fairtrade chocolate, χ^2^(10) = 9.26, *p* = 0.51. Accordingly, none of the variables reliably predicted chocolate choice (see Supplementary Information for the full table of results).

### Discussion

The purpose of this study was to test the correlational findings of Study 1 and 2 experimentally by manipulating both psychological distance and construal level. By manipulating the temporal closeness of climate change, and asking participants to evaluate the stimuli either abstractly or concretely, Study 3 tested the effects of both variables on pro-environmental behavior and measured the corresponding impact on psychological distance and construal level. We compared two hypotheses: (1) that the temporally closest condition (the “past” time horizon) would increase pro-environmental actions, and (2) that the abstract construal condition would be more effective than the concrete construal condition at increasing pro-environmental actions.

The temporal manipulations of psychological distance had an unclear effect on behavior. There was a general trend for engagement in pro-environmental behavior and charity donations to be lower in the past time horizon condition than in the present and future conditions. However, according to CLT, the closest condition should have been the past condition, where the temporal manipulation situated the worst effects of climate change at the date closest to the present (2015). Following this logic, the future should have been the most distant condition, situating the worst effects of climate change in the year 2085. However, the past condition did not lead to greater pro-environmental behavior than the future condition on any of the dependent variables. This finding is counter-intuitive, but it does partially replicate the findings of Schuldt et al. (2018), who found positive responses for the spatially near, temporally distant condition in conservatives — although political views were not a factor here. One possibility is that participants in the past condition did not perceive the final outcome (in the present year) to be as harmful as participants in the other two conditions because it represented the current reality. The videos did not clearly specify the amount of rainfall per year, and so participants may have used the current situation as a baseline.

The construal level manipulations (using the how/why method to induce a concrete vs. abstract mindset) also had no clear effect on pro-environmental behavior, which also means there was little support for the findings of Study 1 and 2. We predicted that the abstract condition would more effective than the concrete condition at increasing pro-environmental behavior. Yet the abstract construal manipulation had no more impact than concrete construal, and did not lead to more pro-environmental behavior than the control condition.

There are potential limitations of the study that merit comment. By combining construal and psychological distance manipulations, we were able to explore their compounded effect, but not their standalone impact on pro-environmental behavior. We expected the compounded effects to produce stronger results from manipulations. However, one possibility is that the combination of construal and distance conditions produced unexpected effects that we cannot disentangle without standalone manipulations against which to compare the results. Nevertheless, our findings support research suggesting that the strength of pre-existing views about climate change makes beliefs about this topic difficult to alter with different frames and mindset inductions (Brügger et al., 2015a; Schuldt et al., 2018). The role of psychological distance and time perspective as predictors of pro-environmental behavior suggest that these views tend to be stable and not easily shifted.

In summary, the manipulation of temporal distance led to similar trends in donation behavior and behavioral intentions, where present and past conditions tended to lead to higher action than future and control conditions. The choice of chocolate showed a trend in favor of CLT, where those in the concrete condition tended to choose fair-trade chocolates more often than those in the abstract condition.

## General Discussion

The aim of this paper was to systematically investigate the relationship between psychological distance and pro-environmental behavior, including the theorized link between construal level and psychological distance. The present study contributes to the literature by testing the theoretical basis for making climate change closer and more personally relevant, and by exploring the separate effects of construal level and psychological distance in the context of climate change on a range of different environmental behaviors. In three studies (two surveys and one experiment) we have demonstrated that, although there is evidence suggesting that psychological distance and construal level are linked to environmental behavior, this connection is complicated, and manipulating these variables does not necessarily lead to greater pro-environmental behavior.

Psychological closeness was a consistent predictor of pro-environmental behavior, explaining variance in individual-level behavior and policy support (Study 1, 2, 3). By contrast, measures of construal level — environmental or general — were less reliable predictors, suggesting that construal level may have limited use for predicting pro-environmental behaviors.

The research clarifies the role of CLT in the context of climate change action–the CLT proposition that concrete and abstract construals shape perceived psychological distance was not supported. We found evidence that both abstract *and* concrete construals explained variance in policy choice (Study 1, 2) and individual behavior and behavioral intentions (Study 1, 2, 3). This suggests that while psychological distance and construal level affect one another and are linked in other contexts, it is not necessarily the case in the context of climate change. One’s perceived psychological distance from climate change appears unrelated to how abstractly one perceives climate change, a finding that complements other studies reporting that representations of climate change are complex and fluid, and at times simultaneously distant and proximal (Brügger and Pidgeon, 2017). The findings identify climate change as an area in which construal level and psychological distance may operate independently rather than interdependently.

Crucially, the inability to affect psychological distance and construal level through experimental manipulations (Study 3) suggests that these constructs are difficult to shift. For instance, there is some evidence to support the idea that construal level may be a stable psychological trait (Darwent, 2012; Sacchi et al., 2016), although this does not explain the lack of correlation between construal level and environmental behavior in Studies 1 and 2.

A growing body of work has shown through multiple methods that it is hard to manipulate psychological distance and construal level to affect pro-environmental action (Brügger et al., 2015b; McDonald et al., 2015; Schuldt et al., 2018). Brügger and Pidgeon (2017) have suggested that variations in individual beliefs, and the focal shifts that these cognitions prompt, can obscure messages designed to frame distance or construal in a particular way. Not only that, but focal shifts that occur as a result of framing manipulations are likely to be temporary and are therefore unlikely to produce lasting change in attitudes or behavior unless the wider context of climate communication changes.

The findings also have implications for the communication of climate change. In particular, the results indicate how to address a lack of public concern toward active engagement with climate change. First, addressing public skepticism about climate change may be more important when seeking support for environmental policies than in the context of individual behaviors and choices. It could be that communication about the imminent and future consequences and impacts of climate change is more effective than showing what has *already* happened (although considering the importance of time perspective, and related temporal discounting literature, the message may need to be more along the lines of “the future is closer than you think”). This is important, as much of climate change communication appears gridlocked on debates about how CO_2_ levels, sea ice, and so on have changed in the past (Pearce et al., 2017). Such discussions not only focus attention away from the forthcoming consequences, they also play into an unnecessary debate about the reality of climate change and validity of climate science.

In light of the findings of the present study, it will be especially important to focus future research on the underlying features of psychological closeness. This study, and others, have found it difficult to shift these views experimentally (Brügger et al., 2015a; McDonald et al., 2015; Brügger and Pidgeon, 2017; Schuldt et al., 2018), and more recent research has focused instead on pinning down the mechanisms underlying psychological distance (Wang et al., 2018). To know that psychological distance is a predictor of pro-environmental behavior is meaningless unless we understand what it entails, to understand how perceptions of closeness are described, and how they manifest, and ultimately, what it means to be psychologically close to climate change.

## Ethics Statement

This study was carried out in accordance with the recommendations of the Human Research Ethics Office of the University of Western Australia with written informed consent from all subjects. All subjects gave written informed consent in accordance with the Declaration of Helsinki. The protocol was approved by the Human Research Ethics Office of the University of Western Australia.

## Author Contributions

SW wrote the initial drafts of the manuscript, and collected data for all studies. SW and MH conducted analyses and MH created the figures. SW, MH, ZL, IW, and CL all contributed to designing the studies, interpreting the findings, and editing the manuscript.

## Conflict of Interest Statement

The authors declare that the research was conducted in the absence of any commercial or financial relationships that could be construed as a potential conflict of interest.

## References

[B1] Arnocky S. Milfont T. L. Nicol J. R. (2013). Time perspective and sustainable behavior: evidence for the distinction between consideration of immediate and future consequences. *Environ. Behav.* 46 556–582. 10.1177/0013916512474987

[B2] Australian Bureau of Statistics (2014). *Population by Age and Sex, Regions of Australia, 2014. Cat. No. 3235.0.* Canberra: Australian Bureau of Statistics.

[B3] Australian Taxation Office (2013). *Taxation Statistics 2012-13: Detailed Tables.* Canberra: Australian Taxation Office.

[B4] Australian Treasury (2013). *Climate Change Mitigation Scenarios: Modelling Report Provided to the Climate Change Authority in support of its Caps and Target review.* Canberra: Commonwealth of Australia Available at: http://www.environment.gov.au/node/35527

[B5] Bar-Anan Y. Liberman N. Trope Y. Algom D. (2007). Automatic processing of psychological distance: evidence from a stroop task. *J. Exp. Psychol. Gen.* 136 610–622. 10.1037/0096-3445.136.4.610 17999574 PMC3161424

[B6] Bashir N. Y. Wilson A. E. Lockwood P. Chasteen A. L. Alisat S. (2014). The time for action is now: subjective temporal proximity enhances pursuit of remote-future goals. *Soc. Cogn.* 32 83–93. 10.1521/soco.2014.32.1.83

[B7] Brügger A. Dessai S. Devine-Wright P. Morton T. A. Pidgeon N. F. (2015a). Psychological responses to the proximity of climate change. *Nat. Clim. Chang.* 5 1031–1035. 10.1038/nclimate2760 23802658

[B8] Brügger A. Morton T. Dessai S. (2015b). Hand in hand: public endorsement of climate change mitigation and adaptation. *PLoS One* 10:e0124843. 10.1371/journal.pone.0124843 25922938 PMC4414563

[B9] Brügger A. Pidgeon N. F. (2017). Spatial framing, existing associations, and climate change beliefs. *Environ. Values* 27 559–584. 10.3197/096327118X15321668325966

[B10] Castro P. (2006). Applying social psychology to the study of environmental concern and environmental worldviews?: contributions from the social representations approach. *J. Commun. Appl. Soc. Psychol.* 16 247–266. 10.1002/casp.864

[B11] Clayton S. Devine-Wright P. Stern P. C. Whitmarsh L. Carrico A. Steg L. (2015). Psychological research and global climate change. *Nat. Clim. Change* 5 640–646. 10.1038/nclimate2622

[B12] Clayton S. Manning C. Hodge C. (2014). *Beyond Storms and Droughts: The Psychological Impacts of Climate Change.* Washington, DC: Psychology Today.

[B13] Darwent K. (2012). *Individual Differences in Travel Across Psychological Distances.* Doctoral dissertation, Ohio State University, Columbus, OH.

[B14] Devine-Wright P. (2013). Think global, act local? The relevance of place attachments and place identities in a climate changed world. *Glob. Environ. Change* 23 61–69. 10.1016/j.gloenvcha.2012.08.003

[B15] Devine-Wright P. Price J. Leviston Z. (2015). My country or my planet? Exploring the influence of multiple place attachments and ideological beliefs upon climate change attitudes and opinions. *Glob. Environ. Change* 30 68–79. 10.1016/j.gloenvcha.2014.10.012

[B16] Faul F. Erdfelder E. Lang A. G. Buchner A. (2007). G^∗^power 3: a flexible statistical power analysis program for the social, behavioral, and biomedical sciences. *Behav. Res. Methods* 39 175–191. 10.3758/BF03193146 17695343

[B17] Fielding K. S. Hornsey M. J. Swim J. K. (2014). Editorial developing a social psychology of climate change. *Eur. J. Soc. Psychol.* 44 413–420. 10.1002/ejsp.2058

[B18] Fujita K. Trope Y. Liberman N. Levin-Sagi M. (2006). Construal levels and self-control. *J. Pers. Soc. Psychol.* 90 351–367. 10.1037/0022-3514.90.3.351 16594824 PMC3153425

[B19] Gifford R. (2011). The dragons of inaction: psychological barriers that limit climate change mitigation and adaptation. *Am. Psychol.* 66 290–302. 10.1037/a0023566 21553954

[B20] Hansen J. Trope Y. (2012). When time flies: how abstract and concrete mental construal affect the perception of time. *J. Exp. Psychol. Gen.* 142 336–347. 10.1037/a0029283 22800441

[B21] Hardisty D. J. Weber E. U. (2009). Discounting future green: money versus the environment. *J. Exp. Psychol. Gen.* 138 329–340. 10.1037/a0016433 19653793

[B22] Hart P. S. Nisbet E. C. (2012). Boomerang effects in science communication: how motivated reasoning and identity cues amplify opinion polarization about climate mitigation policies. *Commun. Res.* 39 701–723. 10.1177/0093650211416646

[B23] Joireman J. Shaffer M. J. Balliet D. Strathman A. (2012). Promotion orientation explains why future-oriented people exercise and eat healthy: evidence from the two-factor consideration of future consequences-14 scale. *Pers. Soc. Psychol. Bull.* 38 1272–1287. 10.1177/0146167212449362 22833533

[B24] Krüger T. Fiedler K. Koch A. S. Alves H. (2014). Response category width as a psychophysical manifestation of construal level and distance. *Pers. Soc. Psychol. Bull.* 40 501–512. 10.1177/0146167213517009 24351751

[B25] Ledgerwood A. Trope Y. Chaiken S. (2010). Flexibility now, consistency later: psychological distance and construal shape evaluative responding. *J. Pers. Soc. Psychol.* 99 32–51. 10.1037/a0019843 20565184 PMC3149789

[B26] Leiserowitz A. (2005). American risk perceptions: is climate change dangerous? *Risk Anal.* 25 1433–1442. 10.1111/j.1540-6261.2005.00690.x 16506973

[B27] Leviston Z. Price J. Malkin S. McCrea R. (2014). *Fourth Annual Survey of Australian Attitudes to Climate Change: Interim Report.* Perth: CSIRO.

[B28] Lewin K. (1951). “Field theory in social science: selected theoretical papers,” in *American Sociological Review*, 1st Edn Vol. 16 ed. Cartwright D. (New York, NY: Harper).

[B29] Liberman N. Trope Y. (2008). The psychology of transcending the here and now. *Science* 322 1201–1205. 10.1126/science.1161958 19023074 PMC2643344

[B30] Liberman N. Trope Y. McCrea S. M. Sherman S. J. (2007). The effect of level of construal on the temporal distance of activity enactment. *J. Exp. Soc. Psychol.* 43 143–149. 10.1016/j.jesp.2005.12.009

[B31] Lima M. L. Castro P. (2005). Cultural theory meets the community: worldviews and local issues. *J. Environ. Psychol.* 25 23–35. 10.1016/j.jenvp.2004.1-4

[B32] Locke E. A. Latham G. P. (2002). Building a practically useful theory of goal setting and task motivation. A 35-year odyssey. *Am. Psychol.* 57 705–717. 10.1037/0003-066X.57.9.705 12237980

[B33] Markle G. L. (2013). Pro-environmental behavior: does it matter how it’s measured? Development and validation of the pro-environmental behavior scale (PEBS). *Hum. Ecol.* 41 905–914. 10.1007/s10745-013-9614-8

[B34] McDonald R. I. Chai H. Y. Newell B. R. (2015). Personal experience and the ‘psychological distance’ of climate change: an integrative review. *J. Environ. Psychol.* 44 109–118. 10.1016/j.jenvp.2015.10.003

[B35] McDonald R. I. Newell B. R. Brewer M. (2013). “Distancing climate change on four dimensions: implications for support for climate change action,” in *Proceedings of the Psychology of Climate Change Symposium* (Sydney: Centre for Excellence in Climate System Science/School of Psychology, University of New South Wales).

[B36] Milfont T. L. Wilson J. Diniz P. (2012). Time perspective and environmental engagement: a meta-analysis. *Int. J. Psychol.* 47 325–334. 10.1080/00207594.2011.647029 22452746

[B37] Moser S. C. (2016). Reflections on climate change communication research and practice in the second decade of the 21st century: what more is there to say? *Wiley Interdiscip. Rev. Clim. Change* 7 345–369. 10.1002/wcc.403

[B38] Owens S. Driffill L. (2008). How to change attitudes and behaviours in the context of energy. *Energy Pol.* 36 4412–4418. 10.1016/j.enpol.2008.09.031

[B39] Pahl S. (2010). “Psychological distance: exploring construal level theory in the context of sustainability,” in *Proceedings of the BPS Seminar Series Psychology of Sustainability*, Cardiff.

[B40] Pahl S. Bauer J. (2013). Overcoming the distance: perspective taking with future humans improves environmental engagement. *Environ. Behav.* 45 155–169. 10.1177/0013916511417618

[B41] Pearce W. Grundmann R. Hulme M. Raman S. Hadley Kershaw E. Tsouvalis J. (2017). A reply to cook and oreskes on climate science consensus messaging. *Environ. Commun.* 11 736–739. 10.1080/17524032.2017.1392109

[B42] Pettigrew T. F. (1958). The measurement and correlates of category width as a cognitive variable. *J. Pers.* 26 532–544. 10.1111/j.1467-6494.1958.tb02350.x

[B43] Price J. C. Walker I. Boschetti F. (2014). Measuring cultural values and beliefs about environment to identify their role in climate change responses. *J. Environ. Psychol.* 37 8–20. 10.1016/j.jenvp.2013.10.001

[B44] Rabinovich A. Morton T. Postmes T. (2010). Time perspective and attitude-behaviour consistency in future-oriented behaviours. *Br. J. Soc. Psychol.* 49(Pt 1), 69–89. 10.1348/014466608X401875 19224678

[B45] Rabinovich A. Morton T. Postmes T. Verplanken B. (2009). Think global, act local: the effect of goal and mindset specificity on willingness to donate to an environmental organization. *J. Environ. Psychol.* 29 391–399. 10.1016/j.jenvp.2009.09.004

[B46] Reese G. Kohlmann F. (2015). Feeling global, acting ethically: global identification and fairtrade consumption. *J. Soc. Psychol.* 155 98–106. 10.1080/00224545.2014.992850 25492312

[B47] Reser J. Bradley G. L. Glendon A. I. Ellul M. C. Callaghan R. (2012). *Public Risk Perceptions, Understandings and Responses to Climate Change and Natural Disasters in Australia, 2010 and 2011.* Gold Coast: National Climate Change Adaptation Research Facility.

[B48] Rickard L. N. Yang Z. J. Schuldt J. P. (2016). Here and now, there and then: how “departure dates” influence climate change engagement. *Glob. Environ. Change* 38 97–107. 10.1016/j.gloenvcha.2016.03.003

[B49] Roh S. McComas K. A. Rickard L. N. Decker D. J. (2015). How motivated reasoning and temporal frames may polarize opinions about wildlife disease risk. *Sci. Commun.* 37 340–370. 10.1177/1075547015575181

[B50] Rosch E. (1999). “Principles of categorization,” in *Concepts: Core Readings*, eds Margolis E. Laurence S. (Cambridge, MA: MIT Press), 189–206.

[B51] Sacchi S. Riva P. Aceto A. (2016). Myopic about climate change: cognitive style, psychological distance, and environmentalism. *J. Exp. Soc. Psychol.* 65 68–73. 10.1016/j.jesp.2016.03.006

[B52] Scannell L. Gifford R. (2011). Personally relevant climate change: the role of place attachment and local versus global message framing in engagement. *Environ. Behav.* 45 60–85. 10.1177/0013916511421196

[B53] Schuldt J. P. Rickard L. N. Yang Z. J. (2018). Does reduced psychological distance increase climate engagement? On the limits of localizing climate change. *J. Environ. Psychol.* 55:147 10.1016/j.jenvp.2018.02.001

[B54] Shwom R. Dan A. Dietz T. (2008). The effects of information and state of residence on climate change policy preferences. *Clim. Change* 90 343–358. 10.1007/s10584-008-9428-7

[B55] Soderberg C. K. Callahan S. P. Kochersberger A. O. Amit E. Ledgerwood A. (2015). The effects of psychological distance on abstraction: two meta-analyses. *Psychol. Bull.* 141 525–548. 10.1037/bul0000005 25420220

[B56] Spence A. Pidgeon N. (2010). Framing and communicating climate change: the effects of distance and outcome frame manipulations. *Glob. Environ. Change* 20 656–667. 10.1016/j.gloenvcha.2010.07.002

[B57] Spence A. Poortinga W. Pidgeon N. (2012). The psychological distance of climate change: psychological distance of climate change. *Risk Anal.* 32 957–972. 10.1111/j.1539-6924.2011.01695.x 21992607

[B58] Steg L. Vlek C. (2009). Encouraging pro-environmental behaviour: an integrative review and research agenda. *J. Environ. Psychol.* 29 309–317. 10.1016/j.jenvp.2008.10.004

[B59] Sundblad E. Biel A. Gärling T. (2011). Cognitive and affective risk judgements related to climate change. *J. Environ. Psychol.* 27 97–106. 10.1016/j.jenvp.2007.01.003

[B60] Todorov A. Goren A. Trope Y. (2007). Probability as a psychological distance: construal and preferences. *J. Exp. Soc. Psychol.* 43 473–482. 10.1016/j.jesp.2006.04.002

[B61] Trope Y. Liberman N. (2003). Temporal construal. *Psychol. Rev.* 110 403–421. 10.1037/0033-295X.110.3.403 12885109

[B62] Trope Y. Liberman N. (2010). Construal-level theory of psychological distance. *Psychol. Rev.* 117 440–463. 10.1037/a0018963 20438233 PMC3152826

[B63] Uzzell D. L. (2000). The psycho-spatial dimension of global environmental problems. *J. Environ. Psychol.* 20 307–318. 10.1006/jevp.2000.0175

[B64] Vallacher R. R. Wegner D. M. (1989). Levels of personal agency: individual variation in action identification. *J. Pers. Soc. Psychol.* 57 660–671. 10.1037/0022-3514.57.4.660 2926623

[B65] Van Boven L. Kane J. McGraw A. P. Dale J. (2010). Feeling close: emotional intensity reduces perceived psychological distance. *J. Pers. Soc. Psychol.* 98 872–885. 10.1037/a0019262 20515244

[B66] Wang S. Leviston Z. Hurlstone M. Lawrence C. Walker I. (2018). Emotions predict policy support: why it matters how people feel about climate change. *Glob. Environ. Change* 50 25–40. 10.1016/j.gloenvcha.2018.03.002

[B67] White K. MacDonnell R. Dahl D. W. (2011). It’s the mind-set that matters: the role of construal level and message framing in influencing consumer efficacy and conservation behaviors. *J. Market. Res.* 48 472–485. 10.1509/jmkr.48.3.472

[B68] Whitmarsh L. (2011). Scepticism and uncertainty about climate change: dimensions, determinants and change over time. *Glob. Environ. Change* 21 690–700. 10.1016/j.gloenvcha.2011.01.016

[B69] Williams L. E. Stein R. Galguera L. (2014). The distinct affective consequences of psychological distance and construal level. *J. Consum. Res.* 40 1123–1138. 10.1086/674212

