# Supplementary material for: Climate Change From a Distance: An Analysis of Construal Level and Psychological Distance From Climate Change
Source: Front Psychol. 2019 Feb 22;10:230. doi: 10.3389/fpsyg.2019.00230 (PMC6395381; doi:10.3389/fpsyg.2019.00230)
Supplement: Supplementary file 1 [file Data_Sheet_1.PDF]

Supplementary Material

Climate change from a distance: An analysis of construal level and psychological distance from climate change

Susie Wang*, **Mark Hurlstone, Zoe Leviston, Iain Walker, Carmen Lawrence**

***Correspondence**: Susie Wang, s.wang@rug.nl

Table of Contents

Study 1 & 2
PD1: Principal Components Analysis 2
PD2: Principal Components Analysis 3
PD2: Estimated reliability 4
Response Category Width: Scale 5
Response Category Width: Principal Components Analysis 7
Behavioural Identification Form: Principal Components Analysis 9
Behavioural Identification Form (Environmental): Pilot Study 9
Behavioural Identification Form: Scale, General and Pro-Environmental 15
Behavioural Identification Form: Scale Reliability 16
Pro-environmental Behaviour: Scale 18
Pro-environmental Behaviour: Regression Analyses 19
Supplementary Analysis: Step-wise model showing contribution of BIF-E to PEB 20
Supplementary Analysis: Mediation analyses from Study 1 and 2 21
Policy Support: Difference between 2014 and 2016 22

Study 3
Donation: Distributions in Study 3 23
Chocolate Choice: Multinomial Logistic Regression 24

PD1: Principal Components Analysis

Table S1 shows component loadings and PCA statistics for the PD1 scale across the first two studies. Loadings are largely consistent between the two studies, and items tend to load on a single component.

Table S1 | Unrotated Principal Components Analysis for PD1.

		Study 1		Study 2	
		1	2	1	2
GEO1	I feel geographically far from the effects of climate change	**0.79**	0.37	**0.76**	-0.26
GEO2	Serious effects of climate change will mostly occur in areas far away from here	**0.66**	**0.59**	**0.62**	-0.53
GEO3	(-)My local area will be affected by climate change	**0.78**	-0.11	**0.79**	0.21
GEO4	(-)Climate change will have consequences for every region, including where I live	**0.77**	-0.31	**0.73**	0.31
SOC1	I don't see myself as someone who will be affected by climate change	**0.85**	0.10	**0.63**	-0.25
SOC2	Serious effects of climate change will mostly affect people who are distant from me	**0.54**	**0.69**	**0.75**	0.28
SOC3	My family and I will be safe from the effects of climate change	**0.79**	0.15	**0.66**	0.32
SOC4	I can identify with victims of climate related disasters	**0.53**	-0.12	**0.80**	-0.16
TEMP1	(-)Climate change is happening now	**0.80**	-0.27	**0.65**	0.36
TEMP2	Reverse.-We will see the serious effects of climate change in my lifetime	**0.66**	0.06	**0.69**	0.32
TEMP3	If climate change is to happen, it will happen in the remote future	**0.82**	-0.29	**0.82**	-0.11
TEMPSOC	Reverse -The region where I live is already experiencing serious effects of climate change	**0.86**	0.02	**0.79**	-0.14
TEMPGEO	Climate change will not change my life, or my family's lives anytime soon	**0.72**	-0.07	**0.79**	-0.21
HYP1	(-)Climate change is virtually certain to affect the world	**0.72**	-0.43	**0.80**	-0.16
HYP2	(-)It is almost certain that climate change will change my life for the worse	**0.82**	-0.25	**0.64**	-0.52
HYP3	It is extremely unlikely that climate change will affect me	**0.89**	0.02	**0.43**	**0.46**
HYPGEO	My local area is very unlikely to be affected by climate change	**0.78**	0.04	**0.81**	-0.15
HYPSOC	It is virtually certain that my family will be safe from the effects of climate change	**0.82**	0.06	**0.67**	**0.43**
Variance explained per component		55.93	6.65	51.54	9.9
Kaiser-Meyer-Olkin Measure of Sampling Adequacy.		0.95		0.94	
Bartlett's Test of Sphericity	Approx. Chi-Square	3213.32		2529.63	
	df	153		153.00	
	Sig.	0.00		0.00	

PD2: Principal Components Analysis

Results for the PCAs for PD2 in both study 1 and 2 are shown in Table S2. The loadings for components is similar between studies, with social and spatial distance loading together, temporal and hypothetical distance loading together, and spatial in between. The relationship is depicted in Figure 1.

Table S2 | Varimax Rotated Component Matrix for PD2.

		Study 1		Study 2	
		1	2	1	2
Social (close)		**0.83**	0.12	**0.82**	0.103
Social (similar)		**0.81**	0.05	**0.782**	0.093
Spatial		**0.65**	**0.50**	**0.775**	0.255
Temporal		0.22	**0.88**	0.337	**0.788**
Hypothetical		0.05	**0.88**	0.024	**0.898**
Variance explained by components		50.463	22.228	48.852	21.372
Kaiser-Meyer-Olkin Measure of Sampling Adequacy.		0.694		0.699	
Bartlett's Test of Sphericity	Approx. Chi-Square	303.596		253.805	
	df	10		10	
	Sig.	<0.001		<0.001	

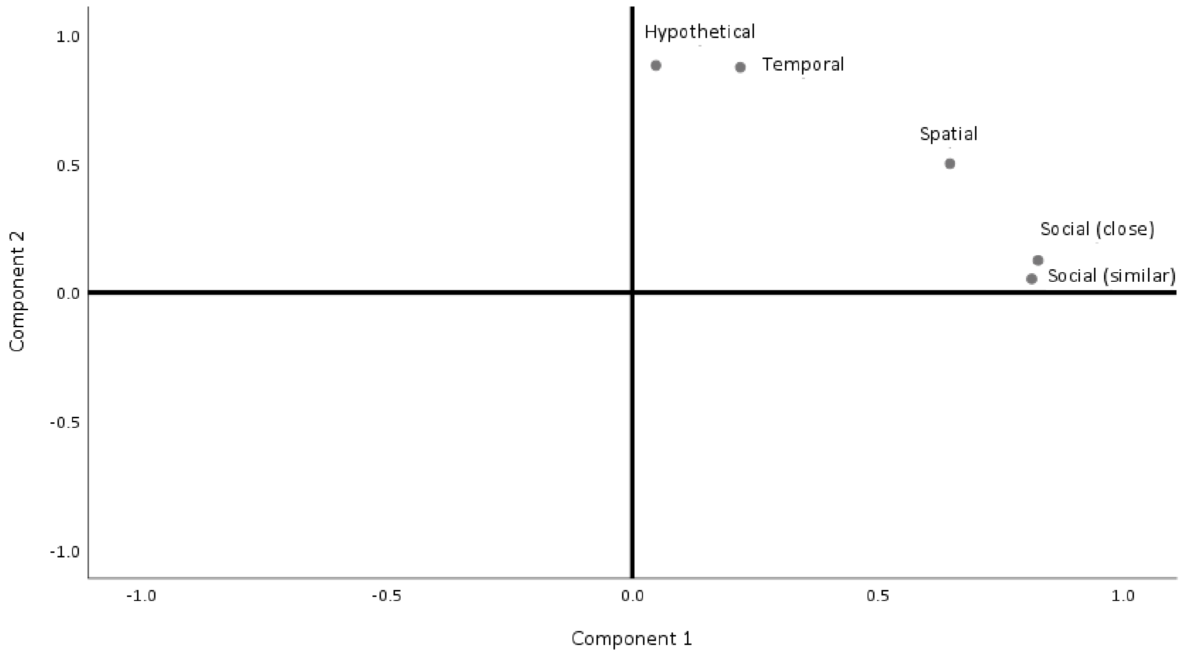

Figure S1 | Dimensions of PD2 in rotated space (Study 1).

PD2: Estimated reliability

As discussed in the manuscript, there is evidence to suggest that the PD1 scale is slightly superior to PD2, but length of scale is an important factor. The PD2 scale contains only 5 items, whereas the PD1 scale contains 18. Using the Spearman-Brown prophecy formula, the estimated reliability of PD2 was calculated at 0.92, almost equivalent to the standardized alpha of PD1. The attenuation formula (Murphy & Davidshofer, 1997) was used to calculate correlations with the Spearman-Brown adjusted reliability. As shown in Table S3, when the internal consistency of PD2 is adjusted, both PD scales have similar correlations with key variables.

Table S3 | Correlations for PD2 (with correction for attenuation).

Variables	PD2	PD1
PD2	1	
PD1	0.821	1
Scepticism	0.700	0.791
Ductile	-0.646	-0.678
Elastic	0.669	0.684
Pro-environmental behaviour	-0.452	-0.504

Response Category Width: Scale

The RCW measures concrete and abstract construal. Items are adapted from Pettigrew (1958) Category Width Estimation Questions. Items are scored 0, 1, or 2, scores increasing with distance from the mean.

Environmental items (Environmental construal)

1. According to the Bureau of Meteorology, in the last 20 years, Perth city has received an average rainfall of 736 millimetres annually. What do you think is:

a) The greatest amount of rain that Perth city received in a single year during this time?

i) 905 mm ii) 2103 mm iii) 793 mm iv) 1224 mm

b) The smallest amount of rain that Perth city received in a single year during this time?

i) 466 mm ii) 105 mm iii) 710 mm iv) 385 mm

2. In the month of July, Perth city received an average of 386 minutes of sunlight. What do you think is:

a) The greatest amount of sunlight received in one day?

i) 558 mins ii) 740 mins iii) 412 mins iv) 657 mins

b) The smallest amount of sunlight received in one day?

i) 0 mins ii) 30 mins iii) 240 mins iv) 301 mins

3. It is estimated that land area needed to support an average Australian lifestyle is 6.6 global hectares. This is equal to 66 000 square metres of land. What do you think is:

a) The amount of land area needed to support the most resource-consuming Australian lifestyle?

i) 70 000 m2 ii) 106 000 m2 ii) 290 000 m2 iv) 540 000 m2

b) The amount of land area needed to support the least resource-consuming Australian lifestyle?

i) 50 000 m2 ii) 26 000 m2 ii) 2 000 m2 iv) 8 000 m2

4. In 2013, an average of 24 900 solar panel systems were installed per state or territory in Australia. What do you think is:

a) The highest number of solar panel systems installed in any Australian state or territory?

i) 70 900 ii) 102 200 iii) 43 000 iv) 31 200

b) The lowest number of solar panel systems installed in any Australian state or territory?

i) 1 020 ii) 19 400 iii) 315 iv) 12 700

5. Between 1971 and 2009, the average yearly rate of ice loss from glaciers around the world was 226 gigatonnes (Gt = 226 billion tonnes of ice per year). What do you think was:

a) The greatest rate of ice loss in any single year?
i) 287 Gt ii) 360 Gt iii) 451 Gt iv) 588 Gt
b) The lowest rate of ice loss in any single year?
i) 52 Gt ii) 91 Gt iii) 133 Gt iv) 205 Gt

6. According to a study of 100 households, the average shower taken consumes 62 litres of water. What do you think is:

a) The most amount of water consumed in a single shower?
i) 71 litres ii) 145 litres iii) 190 litres iv) 232 litres

b) The least amount of water consumed in a single shower?
i) 2 litres ii) 18 litres iii) 35 litres iv) 52 litres

Original Pettigrew Items (General construal)

7. It is estimated that the average width of windows is 86 centimetres. What do you think is:

a) The width of the widest window?
i) 3 460 cm ii) 121 cm iii) 860 cm iv) 205 cm

b) The width of the narrowest window?
ii) 8 cm ii) 27 cm iii) 45 cm iv) 2.5 cm

8. The average muzzle-to-tail length of a sample of 1000 German Shepherd dogs is 104 cm. What do you think

a) Is the length of the longest dog in the sample?
i) 153cm ii) 112 cm iii) 121 cm iv) 137 cm
b) Is the length of the shortest dog in the sample?
ii) 87 cm ii) 50 cm iii) 72 cm iv) 93 cm

9. Ornithologists tell us that the best guess of the average speed of a bird in flight is about 27 km per hour. What do you think is:

a) The speed in flight of the fastest bird?
i) 40km/h ii) 54km/h iii) 117km/h iv) 170 km/h

b) The speed in flight of the slowest bird?
i) 19km/h ii) 16km/h iii) 8km/h iv) 3km/

Response Category Width: Principal Components Analysis

Results from the Varimax rotated PCA for the RCW scale in Study 1 are shown in Table S4. While there were several distinct components, environmental items tended to load on different components than general items. Grey rows indicate environmental items.

Table S4 | Varimax Rotated Component Matrix for RCW scale.

		Components					
		Enviro	General	Enviro	Enviro	Enviro	General
E	The greatest amount of rain that Perth city received in a single year during this time?	0.21	0.08	**0.56**	0.33	0.05	-0.12
E	The smallest amount of rain that Perth city received in a single year during this time?	**0.56**	0.31	0.01	0.09	-0.33	0.04
E	The greatest amount of sunlight received in one day?	0.21	-0.01	0.31	0.31	0.25	0.24
E	The smallest amount of sunlight received in one day?	**0.60**	-0.06	0.05	0.09	0.32	0.09
E	The most amount of water consumed in a single shower?	**0.41**	0.08	**0.63**	0.10	-0.19	0.19
E	The least amount of water consumed in a single shower?	**0.64**	0.25	0.18	0.11	0.06	-0.06
E	The amount of land area needed to support the most resource-consuming lifestyle?	-0.03	0.20	**0.64**	0.01	0.12	0.01
E	The amount of land area needed to support the least resource-consuming Australian lifestyle.	**0.60**	0.06	0.16	-0.03	0.08	0.20
E	The highest number of solar panel systems installed in any Australian state or territory?	-0.11	0.22	0.15	**0.71**	0.07	0.21
E	The lowest number of solar panel systems installed in any Australian state or territory?	**0.49**	0.24	-0.09	0.22	**0.41**	-0.06
E	The greatest rate of ice loss in any single year?	0.32	-0.07	0.06	**0.76**	-0.04	-0.09
E	The lowest rate of ice loss in any single year?	0.15	0.13	0.09	0.01	**0.79**	0.04
G	The width of the widest window?	0.00	**0.56**	0.24	0.35	0.14	0.23
G	The width of the narrowest window?	0.20	**0.64**	0.12	0.08	0.00	-0.04
G	The length of the longest dog in the sample?	0.06	0.02	0.21	-0.03	0.05	**0.85**
G	The length of the shortest dog in the sample?	0.17	0.27	-0.33	0.24	-0.03	**0.71**
G	The speed in flight of the fastest bird?	0.03	**0.55**	0.32	0.04	-0.05	0.11
G	The speed in flight of the slowest bird?	0.15	**0.74**	-0.09	-0.08	0.17	0.07
	Variance explained	22.50	8.01	7.24	6.80	5.90	5.59

Extraction Method: Principal Component Analysis.
Rotation Method: Varimax with Kaiser Normalization.
a. Rotation converged in 9 iterations.

Results from the Varimax rotated PCA for Study 2 are shown in Table S5 below. A similar trend is shown.

Table S5 | Varimax Rotated Component Matrix for RCW scale (Study 1).

		Components				
		Enviro	General	Enviro	General	General
E	The greatest amount of rain that Perth city received in a single year during this time?	0.10	0.26	**0.61**	-0.14	0.00
E	The smallest amount of rain that Perth city received in a single year during this time?	**0.54**	0.16	-0.01	0.04	0.17
E	The greatest amount of sunlight received in one day?	-0.02	-0.08	**0.51**	0.14	**0.48**
E	The smallest amount of sunlight received in one day?	**0.61**	-0.08	0.08	0.10	-0.09
E	The most amount of water consumed in a single shower?	0.38	0.19	**0.46**	0.26	-0.29
E	The least amount of water consumed in a single shower?	**0.48**	0.37	0.10	-0.04	0.26
E	The amount of land area needed to support the most resource-consuming lifestyle?	0.06	0.09	**0.72**	0.09	0.03
E	The amount of land area needed to support the least resource-consuming Australian lifestyle.	**0.57**	0.12	0.11	0.14	0.10
E	The highest number of solar panel systems installed in any Australian state or territory?	0.38	**0.48**	0.22	-0.18	-0.03
E	The lowest number of solar panel systems installed in any Australian state or territory?	**0.57**	-0.14	0.01	0.06	**0.46**
E	The greatest rate of ice loss in any single year?	0.38	0.04	0.39	0.31	-0.11
E	The lowest rate of ice loss in any single year?	0.19	0.02	-0.16	-0.04	**0.75**
G	The width of the widest window?	0.04	0.13	0.20	**0.77**	0.05
G	The width of the narrowest window?	0.21	0.18	-0.14	**0.78**	0.11
G	The length of the longest dog in the sample?	-0.02	**0.75**	0.06	0.22	-0.04
G	The length of the shortest dog in the sample?	-0.11	**0.58**	0.08	0.09	**0.45**
G	The speed in flight of the fastest bird?	0.18	**0.54**	0.15	0.21	-0.02
G	The speed in flight of the slowest bird?	0.14	0.24	0.23	0.32	**0.48**
	Variance explained	21.20	8.91	7.63	6.89	5.79

Extraction Method: Principal Component Analysis.
Rotation Method: Varimax with Kaiser Normalization.
a. Rotation converged in 13 iterations.
E: Environmental items
G: General items

Behavioural Identification Form: Principal Components Analysis

Results for the PCA for the BIF scale in Study 2 are shown in Table S6. These results can be compared with those of the pilot study (next page), where we initially tested this scale with both general and environmental items. The BIF item loadings fell on three components, apparently distinguished by the nature of the behaviours described, rather than their (lack of) environmental content. For instance, general items such as "greeting someone", "resisting temptation", loaded on the same component as the environmental item "using canvas bags for shopping", while environmental items such as "recycling", "installing solar panels", loaded on a separate component, with general behaviours such as "measuring a room for carpeting".

Table S6 | Varimax Rotated Component Matrix.

		Component		
		1	2	3
G	Washing clothes	0.04	0.08	0.01
G	Growing a garden	-0.09	0.10	0.21
G	Measuring a room for carpeting	**0.70**	0.05	0.18
G	Cleaning the house	**0.35**	0.05	0.07
G	Painting a room	0.17	0.12	-0.11
G	Caring for houseplants	0.09	**0.34**	**0.52**
G	Voting	0.19	0.00	0.01
G	Taking a test	-0.01	0.19	0.13
G	Greeting someone	0.22	**0.68**	0.06
G	Resisting temptation	-0.06	**0.64**	0.20
G	Eating	-0.04	**0.32**	-0.07
G	Having a cavity filled	0.24	**0.32**	0.09
E	Turning off lights in empty rooms	0.30	-0.20	0.27
E	Carpooling	0.10	-0.06	**0.73**
E	Composting	-0.07	0.12	0.30
E	Littering	0.12	0.11	**0.51**
E	Recycling	**0.58**	0.11	0.02
E	Buying local products	**0.43**	0.00	0.06
E	Taking public transport	0.19	0.29	**0.50**
E	Installing solar panels	**0.53**	0.28	0.20
E	Using canvas bags for shopping	**0.37**	**0.62**	-0.06
E	Using a shower timer	0.18	0.10	0.29
	Variance explained	18.969	7.505	5.98
Kaiser-Meyer-Olkin Measure of Sampling Adequacy.			0.773	
Bartlett's Test of Sphericity	Approx. Chi-Square		710.99	
	df		231	
	Sig.		<0.001	

Rotation Method: Varimax with Kaiser Normalization. Rotation converged in 13 iterations

Behavioural Identification Form (Environmental): Pilot Study

Contrary to published research, there was no relationship between construal level and psychological distance in the context of climate change and pro-environmental action. A possible explanation for the lack of relationship may be that the conventional measure of construal, namely the BIF, did not contain items specifically addressing the construal of climate change, or pro-environmental actions. On the other hand, psychological distance items directly addressed the topic. Consequently, we conducted a follow-up study to examine the relationship between construal and psychological distance using a modified version of the Vallacher and Wegner (1989) scale. The objective of the follow-up study was to compare psychological distance from climate change, with construal of climate change, measured by BIF items that addressed environmental behavioural construal.

Method

Participants and design

The sample consisted of 494 first year psychology students (282 female), who opted to complete a survey as part of their psychology unit. The average age of participants was 20 years (range = 18-42).

Materials and procedure

Participants were given a paper-based survey that included two questionnaires: a psychological distance scale, and a construal level scale. A short form psychological distance scale was constructed from the 7 highest loading items from the PD1 psychological distance scale, shown in the Principal Components Analysis in Study 1. The scale had good internal consistency (α =.76), though due to error, one low-loading item was incorrectly included, and one high-loading item was omitted ("My local area is very unlikely to be affected by climate change" component loading 0.784, was included instead of "It is unlikely that I will be affected by climate change", component loading 0.886).

The modified BIF scale consisted of 22 items, 11 items from the original scale that did not address environmental issues, and an additional 11 items that formed the environmental subscale. The included one of the original items addressing a pro-environmental action, and 10 new items describing other pro-environmental behaviours. Participants were asked to select either a concrete or an abstract description for each action. For instance, the behavior "carpooling" was described as "sharing transportation with others" (concrete), or "reducing the

number of cars on the road" (abstract). As with the original scale, concrete construals were coded "0", and abstract construals were coded "1". The internal consistency of the scale was 0.76.

Results

No outliers were evident, and the data were normally distributed. Descriptive statistics are shown in Table 7. The BIF variable is presented as the proportion of total responses that were abstract.

Table S7 | Means, ranges and standard deviations.

Variables	Lower (absolute)	Mean	Upper (absolute)	SD
BIF total	0.00 (0)	0.475	1.00 (1)	0.192
BIF-E	0.00 (0)	0.420	1.00 (1)	0.210
BIF-G	0.00 (0)	0.529	1.00 (1)	0.236
PD1	1.00 (1)	2.539	4.57 (5)	0.632

A PCA was conducted, with adequate sampling (KMO = 0.811), and Bartlett's test showed that the null hypothesis can be rejected, χ^2 (231) = 1129.578, $p < 0.001$. Though initial analysis extracted seven factors with eigenvalues greater than 1, the majority of items load on one-component, which accounted for 16.82% of variance (Table S8). Loadings on the first component occurred across environmental and general items; there was no distinction between the two sets of items. This indicates that the environmental BIF items are not entirely distinct from the original, general BIF items.

The correlations between variables are shown in Table S9. General and environmental construal were significantly, and moderately correlated. This suggests that general construal accounts for a modest amount of variance in environmental construal. Psychological distance shows a small negative correlation with environmental construal, suggesting that as one's

Table S8 | Unrotated Component Matrix for BIF (General and Environmental).

		Component						
		1	2	3	4	5	6	7
Environmental scale	Growing a garden	.362	.201	-.230	-.049	-.433	.024	-.440
	Buying Local Products	.293	-.264	-.402	.282	.205	.319	.034
	Littering	**.522**	-.122	-.188	.121	-.276	-.118	-.117
	Solar Panels	**.408**	-.456	-.003	-.060	.067	-.145	-.023
	Turning off lights	**.504**	-.007	.066	-.034	.081	.216	.178
	Public Transport	**.405**	-.281	-.358	-.007	.336	-.076	-.109
	Canvas bags	**.430**	-.345	.050	-.006	-.395	.177	.297
	Composting	**.509**	-.131	-.118	-.129	-.148	-.038	.164
	Carpooling	.389	-.253	.174	-.041	.251	-.089	.332
	Recycling	**.455**	-.286	.124	-.012	-.262	.032	-.177
	Shower timer	.312	-.124	**.499**	.051	.251	.347	-.281
	Houseplants	.385	.196	-.404	.035	.308	.306	-.164
Original scale	Washing Clothes	.326	.383	.023	-.057	-.193	**.464**	.351
	Carpeting	.339	.377	-.006	.346	-.023	-.106	.298
	Cleaning house	.322	**.424**	-.150	-.326	.168	-.251	.238
	Painting room	**.437**	.328	-.184	**.468**	.061	-.176	-.131
	Voting	.246	-.093	.348	**.562**	.030	-.339	.112
	Taking a test	**.456**	.094	.208	-.168	-.098	.019	.025
	Greeting someone	**.557**	.125	.018	-.142	-.109	-.269	-.140
	Resisting temptation	**.434**	.010	.036	-.470	.211	-.224	-.048
	Eating	.264	.247	**.494**	.018	.083	.117	-.266
	Filling cavity	**.474**	.183	.241	-.010	.170	.026	-.084

Extraction Method: Principal Component Analysis.

psychological distance from climate change increases, construal of climate change becomes slightly more concrete.

Discussion and Conclusion

The aim of this pilot study was to examine the relationship between psychological distance and construal level when both are measured using scales addressing climate change and pro-environmental construals.

Firstly, component loadings showed no clear distinction between pro-environmental and general items, which indicates that the pro-environmental BIF items are not measuring an entirely different construct from the general BIF items. Similarly, the correlation shows a

moderate, but significant correlation between the two variables. This suggests that while pro-environmental and general construals of behaviours are distinct, of the same kind.

The results of this study partially replicated the findings of Study 1 in that the general BIF was again unrelated to psychological distance. There was no apparent correlation between the construal of general items and psychological distance from climate change. However, contrary to previous research, we found a negative association between construal level of pro-environmental behaviours and psychological distance from climate change. There was a small but significant correlation between pro-environmental BIF and psychological distance. This means that as distance from climate change increases, construal of pro-environmental actions becomes slightly less abstract, and more concrete. One is *less* likely to make environmental, abstract attributions for behaviours such as carpooling, recycling, and reducing electricity use if one feels distant from climate change. This is a notable finding because it suggests the opposite relationship to that hypothesised by the CLT literature. According to a large body of work, as psychological distance increases, so too does the abstractness of construal (Soderberg et al., 2014).

Table S9 | Correlations.

		Environmental Abstractness	General Abstractness	Psychological distance
Environmental Abstractness	*R*	1	.531**	-.211**
	p		.000	.000
	N	495	495	455
General Abstractness	*R*		1	-.015
	p			.482
	N			455

**. Correlation is significant at the 0.01 level (2-tailed).
R corresponds to Pearson's correlation coefficient

However notable, it is important that this be a tentative conclusion only, due to limitations in measurement instruments. Firstly, it could be argued that the pro-environmental BIF conflates "abstract" construal with environmental attributions. For instance, participants could have chosen to describe the behaviour "turning off lights in empty rooms" as concrete, "remembering to turn off switches", which describes the means by which lights may be turned off. The behaviour may also be described abstractly, as "reducing energy use", which focuses on the goal, and more abstract function of the behaviour. The latter is indeed more abstract, but unlike the concrete description, it also acknowledges the environmental purpose of the behaviour. Similarly, the item "buying local products" had a concrete construal that was

descriptive, “shopping at a farmer’s market”, and an abstract construal that referenced an environmental purpose, “reducing food miles and carbon footprint”.

The possible conflation of environmental attributions and abstract construal is important because it may explain the unexpected negative association between psychological distance and construal level. If one feels that climate change is distant, one will be more likely to reject environmental attributions for behaviours. However, component loadings from the PCA does not suggest a large difference in the pro-environmental compared to general BIF items. A second limitation is that construals of pro-environmental behaviours are not the same as construals of climate change. Due to the format of the BIF, as a scale focused on behaviours, it was difficult to construct behaviours relating to climate change directly. Instead, the items were created to address pro-environmental actions.

Given these limitations, we are not arguing that psychological distance increases with concreteness in the context of climate change, rather, that the relationship between the variables is not easily predicted, and an increase in distance does not always correspond with an increase in abstractness. For the issue of climate change, the assumption that the concreteness and abstractness with which individuals construe climate change shapes their perceived psychological distance does not hold. This finding corroborates those reported in the main study, and substantiates the conclusion that climate change is an area in which the relationship between construal level and psychological distance can, and do, operate independently.

Behavioural Identification Form: Scale, General and Pro-Environmental

Below is the BIF scale used in Study 2 & 3, modified to include environmental items, and including the original instructions.

Instructions: Any behaviour can be identified in many ways. For example, one person might describe a behaviour as "typing a paper," while another might describe the behaviour as "pushing keys". We are interested in your personal preferences for how a number of different behaviours should be described. Your task is to choose the identification, a) or b), that best describes the behaviour for you.

1. Washing clothes
a) Removing odours
b) Putting clothes in the machine

2. Buying local products
a) Shopping at a farmer's market
b) Reducing food miles and carbon footprint

3. Littering
a) Dropping rubbish on the ground
b) Spoiling the environment

4. Measuring a room for carpeting
a) Getting ready to remodel
b) Using a tape measure

5. Installing solar panels
a) Generating your own electricity
b) Producing clean energy

6. Turning off lights in empty rooms
a) Remembering to turn off switches
b) Reducing energy use

7. Cleaning the house
a) Showing one's cleanliness
b) Vacuuming the floor

8. Painting a room
a) Applying brush strokes
b) Making the room look fresh

9. Taking public transport
a) Catching a bus or train
b) Travelling in an energy efficient way

10. Caring for houseplants
a) Watering plants
b) Making the room look nice

11. Growing a garden
a) Planting seeds
b) Getting fresh vegetables

12. Using canvas bags for shopping
a) Reusing bags
b) Reducing waste

13. Voting
a) Influencing the election
b) Marking a ballot

14. Composting
a) Decomposing food scraps
b) Gardening organically

15. Taking a test
a) Answering questions
b) Demonstrating one's knowledge

16. Carpooling
a) Sharing transportation with others
b) Reducing the number of cars on the road

17. Recycling
a) Placing materials in a bin for re-use
b) Preventing waste

18. Greeting someone
a) Saying hello
b) Showing friendliness

19. Resisting temptation
a) Saying "no"
b) Showing moral courage

20. Eating
a) Getting nutrition
b) Chewing and swallowing

21. Having a cavity filled
a) Protecting your teeth
b) Going to the dentist

22. Using a shower timer
a) Reducing water use
b) Having shorter showers

Behavioural Identification Form: Scale Reliability

In Study 3, there was no significant mean difference in BIF scores between conditions (BIF-E: F (6) = 0.848, p = 0.534; BIF-G: F (6) = 0.260, p = 0.955). However, the scale was only moderately internally consistent (BIF α=0.514), with BIF-E (α=0.327) showing less consistency than BIF-G (α=0.526).

As shown in Table S10, the low alpha was not a result of any item in particular. Table S11 looks at the environmental items specifically, and we can draw a similar conclusion – the exclusion of any particular item would not lead to an increase in Cronbach's alpha.

Table S10 | Study 3 BIF Item Reliability Statistics.

		If item dropped Cronbach's α
BIF1	Washing clothes	0.499
BIF2	Growing a garden	0.513
BIF3	Turning off lights in empty rooms	0.531
BIF4	Measuring a room for carpeting	0.496
BIF5	Carpooling	0.526
BIF6	Composting	0.520
BIF7	Cleaning the house	0.495
BIF8	Littering	0.511
BIF9	Recycling	0.505
BIF10	Buying local products	0.533
BIF11	Painting a room	0.519
BIF12	Caring for houseplants	0.528
BIF13	Voting	0.491
BIF14	Taking public transport	0.488
BIF15	Taking a test	0.465
BIF16	Greeting someone	0.486
BIF17	Resisting temptation	0.475
BIF18	Installing solar panels	0.487
BIF19	Eating	0.499
BIF20	Using canvas bags for shopping	0.495
BIF21	Having a cavity filled	0.495
BIF22	Using a shower timer	0.487

Table S11| Environmental BIF Item Reliability Statistics.

		If item dropped Cronbach's α
BIF3	Turning off lights in empty rooms	0.267
BIF5	Carpooling	0.262
BIF6	Composting	0.231
BIF8	Littering	0.228
BIF9	Recycling	0.267
BIF14	Taking public transport	0.290
BIF18	Installing solar panels	0.270
BIF20	Using canvas bags for shopping	0.260
BIF22	Using a shower timer	0.282

As shown in Table S12, the alpha scores were relatively stable in all experimental conditions, except the control condition, where internal consistency was negative or low.

Table S12 | Cronbach's alpha for BIF Environmental, per condition.

Condition	N	Total Cronbach's α	α for BIF-E	α for BIF-G
Control	46	-0.13	-0.57	0.06
Past / Concrete	47	0.56	0.44	0.48
Present / Concrete	43	0.31	0.02	0.52
Future / Concrete	47	0.58	0.37	0.62
Past / Abstract	44	0.61	0.42	0.57
Present / Abstract	48	0.60	0.51	0.62
Future / Abstract	44	0.61	0.28	0.59

frontiers

Pro-environmental Behaviour: Scale

This scale measures willingness to sacrifice money, time, effort and social relationships for pro-environmental gains. The scale was based on Leviston et al. (2014) and was designed to cover different types of pro-environmental behaviour comprehensively (e.g. the domains of food, transport, energy conservation, activism) based on various factor analyses of different kinds of behaviour (Markle, 2013; Steg & Vlek, 2009).

Instructions: We're interested to know about the real choices that people make in day-to-day life. There are no right or wrong answers. How likely are you to do the following things?

1	2	3	4	5
**Very unlikely**	**Unlikely**	**Undecided**	**Likely**	**Very likely**

Buy a regular shampoo for $5, compared to an eco-friendly shampoo for $10? ____

Buy energy efficient LED light-bulbs for $18 each, compared to regular halogen light bulbs for $5 each? ____

Pay $1000 for a four-star fridge, compared to $500 for a two-star fridge? ____

Buy organic, local vegetables at an average cost of $30 per week, compared to imported vegetables at an average cost of $20 per week? ____

Pay an extra $5 to carbon offset your flight, compared to paying only the standard fare? ____

Catch a bus somewhere for 20 minutes, rather than driving there for 5 minutes? ____

Walk to the shops for 15 minutes, rather than driving there for 3 minutes? ____

Set an automatic sprinkler to water your garden, compared to taking half an hour to water your garden by hand? ____

Use a dryer, rather than drying clothes on a line? ____

Use the plastic bags at the grocery store, rather than bring your own bags? ____

Turn a heater on if you're cold, rather than putting on warmer clothes? ____

Drop rubbish on the ground, rather than hold onto it until there is a bin nearby? ____

Buy herbs and vegetables, compared to growing some or all of your herbs and vegetables yourself? ____

Throw recyclable materials in a general waste bin, rather than hold onto recyclable material until there is a recycle bin nearby ____

Leave appliances turned on at power outlets on the wall, rather than switch off appliances at the wall when not in use? ____

Keep quiet about unsustainable and energy-wasting practices in your workplace, rather than suggest ways to improve these practices? ____

Keep quiet about a friend's unsustainable behaviours, rather than point out the unsustainable behaviours and suggest alternatives? ____

Pro-environmental Behaviour: Regression Analyses

Variance inflation factor scores

Table 13 shows the VIF scores between variables for Studies 1 & 2. The exclusion criteria for variables in the final regression models were determined based on centrality of the variable to the central research question (PD, CL variables prioritised), the internal consistency of the scales, and whether the model significantly improved with the inclusion or exclusion of a vari able.

Table S13 | Variance Inflation Factor scores for Regressions.

	Study 1	Study 2
Gender	1.23	1.21
Age	1.25	1.15
Politics	1.30	1.19
Income	1.15	1.19
Belief	**2.93**	**2.31**
Scepticism	**5.11**	**3.13**
Behavioural control	1.29	1.79
Ductile	**3.01**	**1.86**
Elastic	**3.06**	**2.37**
PD1	**4.27**	1.30
PD2	**2.58**	1.61
BIF-G	1.14	1.76
BIF-E	--	**1.99**
RCW-G	1.43	1.53
RCW-E	1.40	1.49
Time perspective	**2.07**	1.67
Place attachment	1.29	1.20

Supplementary Analysis: Step-wise model showing contribution of BIF-E to PEB

Table S14 shows the contribution of BIF-E to the model predicting pro-environmental behaviour in Study 2. The additional step contains only BIF-E and shows a significant increase in variance explained by the additional variable.

Table S14 |Step-wise Regression showing effect of BIF-E to Study 2 Pro-environmental Behaviour.

	Pro-environmental behaviour β (*SE*) (Step 1)	(Step 2)
Gender (M)	-0.23$^{.}$ (0.12)	-0.26* (0.12)
Age	0.01** (0.00)	0.01** (0.00)
Politics	-0.05 (0.04)	-0.04 (0.04)
Income	-0.06* (0.03)	-0.04 (0.03)
Belief	-0.16 (0.16)	-0.19 (0.16)
Scepticism	-0.14 (0.09)	-0.14 (0.09)
Behavioural control	0.12$^{.}$ (0.07)	0.10 (0.07)
PD1	-0.11$^{.}$ (0.06)	-0.09 (0.06)
BIF-G	0.08 (0.06)	-0.04 (0.07)
BIF-E		0.22** (0.08)
RCW-G	-0.01 (0.07)	0.02 (0.07)
RCW-E	-0.07 (0.07)	-0.06 (0.07)
Time Perspective	0.29** (0.07)	0.25** (0.07)
Place Attachment	0.10 (0.06)	0.10 (0.06)
Constant	0.42 (0.30)	0.41 (0.30)
Observations	213	213
R^2	0.38	0.41
Adjusted R^2	0.34	0.36
Residual Std. Error	0.82 (df = 199)	0.80 (df = 199)
Model Comparison		
Sums of Squares		*-5.23*
F		*8.12*
p		*<0.01*

Note: . p<0.1; *p<0.05; **p<0.01

Supplementary Analysis: Mediation analyses from Study 1 and 2

As PD1 and scepticism showed a high amount of shared variance across both correlational studies, the additional mediation analyses below show the extent of their overlap. The various analyses show that the direction of the mediation differs by study and dependent variable, and that the proportion mediated varies from 45% to 100%. The analysis for Study 2 PEB was not included because neither of the variables were significant predictors of pro-environmental behaviour in that study.

Table S15 | Mediation analyses for PD1 and Scepticism in Study 1 & 2.

Study 1	PEB Analysis: PD1 mediates scepticism				
	Estimate	95% CI Lower	95% CI Upper	p-value	
ACME	-0.16	-0.29	-0.02	0.02	*
ADE	-0.19	-0.44	0.02	0.09	.
Total effect	-0.35	-0.55	-0.16	<0.001	***
Prop. Mediated	0.45	0.07	1.12	0.02	*
Study 1	Policy Analysis: Scepticism mediates PD1				
	Estimate	95% CI Lower	95% CI Upper	p-value	
ACME	-0.33	-0.48	-0.19	<0.001	**
ADE	-0.02	-0.15	0.18	0.81	.
Total effect	-0.31	-0.47	-0.14	<0.001	**
Prop. Mediated	1.06	-0.64	2.03	<0.001	**
Study 2	Policy analysis: Scepticism mediates PD1				
	Estimate	95% CI Lower	95% CI Upper	p-value	
ACME	-0.10	-0.18	-0.04	<0.01	**
ADE	-0.02	-0.15	0.10	0.734	
Total effect	-0.12	-0.27	0.01	0.084	.
Prop. Mediated	0.83	-1.95	4.70	0.086	.

Note: The analyses for PEB Study 2 were not included because neither PD1 nor scepticism were significant predictors in the model

Policy Support: Difference between 2014 and 2016

There may have been external changes that affected the results between two studies, and particularly the perception of climate change policies. Study 1 was conducted in 2014, and Study 2 in 2016 – in 2014, a climate change policy was being changed. An Emissions Trading Scheme climate policy was being repealed and replaced with new policy, and so the issue was at the forefront of many political and policy discussions. By 2016, this was no longer the case.

We can see from Figure 2 showing support for policy between the two samples that the earlier sample was much more supportive of stronger emissions reduction policies, although this was not a significant difference, $\chi^2 = 3.82$, $p = 0.43$.

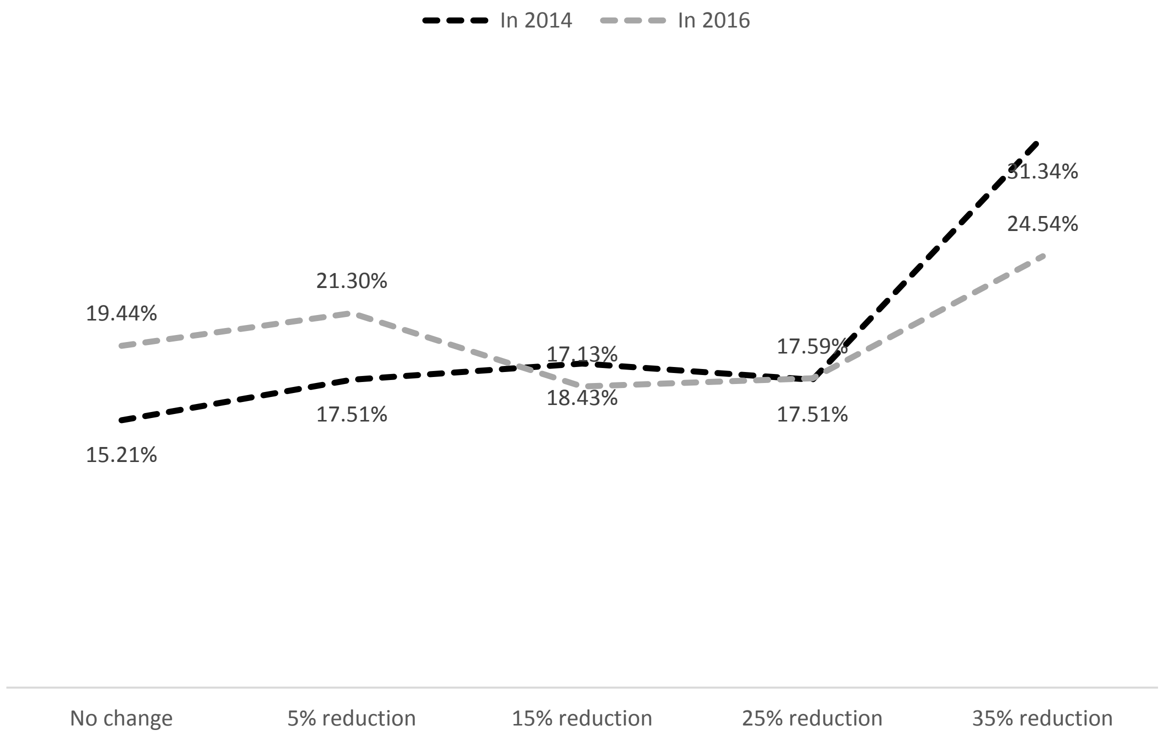

Figure 2 | Percentage of support for emissions reduction policy from Study 1 (2014) and Study 2 (2016).

Donation: Distributions in Study 3

Figure 3 shows the distribution of Donations to Gondwana Link, measured as an explicit pro-environmental behaviour. The distribution of donations was not linear, but instead peaked at salient numbers.

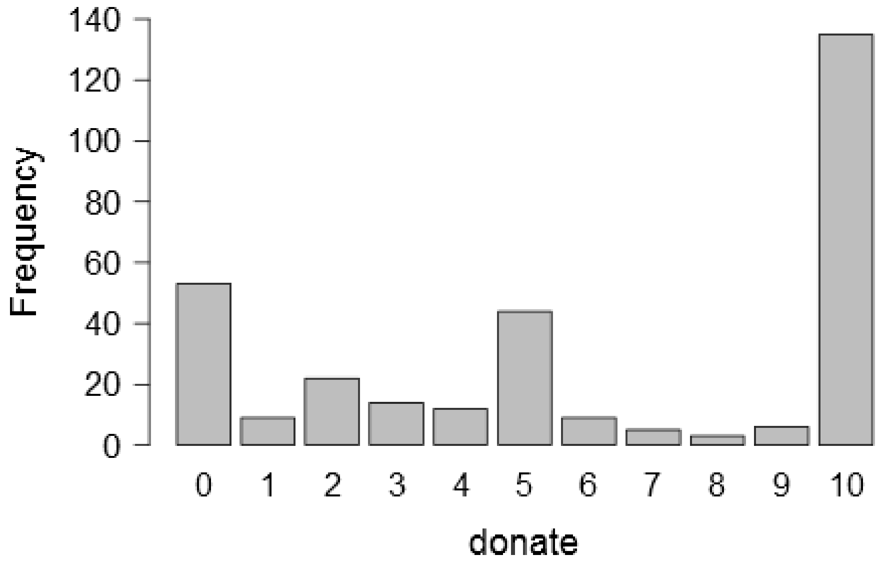

Figure 3 | Distribution of donations to Gondwana-Link in Study 3.

Chocolate Choice: Multinomial Logistic Regression

Table S16 shows the full results of the reported multinomial logistic regression predicting chocolate choice in Study 3.

Table 16 | Results of the multinomial logistic regression analysis of chocolate choice in Study 3.

		95% CI for Odds ratio		
	β (*SE*)	*Lower*	*Odds ratio*	*Upper*
Fairtrade vs. No Chocolate				
Intercept	0.97 (0.42) *			
Construal level	0.08 (0.63)	0.31	1.08	3.72
Time horizon: Present	-0.53 (0.55)	0.20	0.59	1.71
Time horizon: Future	0.08 (0.60)	0.33	1.09	3.56
Construal level × time horizon: Present	0.41 (0.87)	0.27	1.51	8.30
Construal level × time horizon: Future	0.03 (0.90)	0.18	1.03	5.99
Fairtrade vs. Non-Fairtrade				
Intercept	0.63 (0.44)			
Construal level	0.58 (0.64)	0.51	1.78	6.22
Time horizon: Present	-0.71 (0.59)	0.15	0.49	1.57
Time horizon: Future	0.06 (0.64)	0.31	1.07	3.71
Construal level × time horizon: Present	0.71 (0.89)	0.36	2.03	11.54
Construal level × time horizon: Future	-0.06 (0.92)	0.16	0.94	5.65

Note—Likelihood ratio test = $\chi^2(2) = 9.2614$, $p = 0.51$; McFadden $R^2 = 0.02$; * = 0.05.
